# Immunoinformatics-Guided Design, Immunological Evaluation, and Macrophage Transcriptomic Profiling of a Multi-Epitope Vaccine Candidate Against *Mycobacterium avium* subsp. *paratuberculosis*

**DOI:** 10.3390/microorganisms14102188

**Published:** 2026-09-30

**Authors:** Zhijie Luo, Zengke Wei, Jialuo Xu, Baofeng Hao, Wenxue Li, Yue Li, Chunyao Cheng, Xia Zhou, Taishan Fan, Hui Zhang, Jia Guo, Zhen Wang

**Affiliations:** 1 College of Animal Science and Technology, Shihezi University, Shihezi 832003, China; 20232013033@stu.shzu.edu.cn (Z.L.);; 2 Agriculture and Rural Affairs Bureau of Qapqal Xibe Autonomous County, Ili 835300, China

**Keywords:** *Mycobacterium avium* subsp. *paratuberculosis*, Johne’s disease, multi-epitope vaccine, immunoinformatics, recombinant protein, immunogenicity, macrophages, transcriptomics

## Abstract

*Mycobacterium avium* subsp. *paratuberculosis* (MAP) causes Johne’s disease in ruminants and is difficult to control once persistent infection is established. We designed a MAP multi-epitope vaccine candidate by screening conserved proteins and assembling predicted B-cell, MHC class I-restricted cytotoxic T lymphocyte (CTL), and MHC class II-restricted helper T lymphocyte (HTL) epitopes into a recombinant construct. The construct was examined by physicochemical analysis, structural modeling, molecular docking, and immune simulation, then expressed in *Escherichia coli* and tested in BALB/c mice as a recombinant protein without external adjuvant or as an exploratory MYY-containing formulation, alongside PBS and inactivated-MAP controls. RAW264.7 macrophages were stimulated with the purified recombinant protein for RNA sequencing. Seven proteins were retained as antigen sources, and the final construct contained seven HTL epitopes, five B-cell epitopes, and ten CTL epitopes. Immunized mice produced antigen-specific IgG, IgG1, and IgG2a, and antigen-restimulated splenocytes secreted higher levels of IFN-γ, IL-2, TNF-α, and IL-17. Vaccine-induced antibodies recognized both the recombinant protein and components present in MAP lysate while showing limited reactivity toward the bovine tuberculosis diagnostic antigen PPD, and sequence analysis predicted no substantial similarity between the construct and the diagnostic antigens ESAT-6, CFP-10, MPB70, and MPB83, supporting potential DIVA compatibility. In macrophages, recombinant-protein stimulation changed gene-expression profiles and enriched pathways related to cytokine-cytokine receptor interaction, NF-κB, TNF, IL-17, Toll-like receptor, and NOD-like receptor signaling. The construct was immunogenic in mice; the exploratory MYY-containing formulation showed numerically higher immune-response readouts in this experimental setting, although these differences were not statistically significant. This adjuvant-associated observation requires further validation, and protection against MAP infection still needs to be tested in challenge experiments and target animals.

## 1. Introduction

### 1.1. Mycobacterium avium subsp. paratuberculosis Infection and the Challenge of Johne’s Disease

*Mycobacterium avium* subsp. *paratuberculosis* (*MAP*) is a slow-growing intracellular member of the Mycobacterium avium complex and the causative organism of Johne’s disease (JD). This chronic granulomatous enteritis mainly affects ruminants and is associated with a long incubation period, progressive intestinal inflammation, weight loss, reduced milk yield, impaired reproduction, and premature culling. Together, these features make MAP infection a persistent and costly problem in livestock production [1,2].

A central difficulty in MAP control is the organism’s ability to persist within macrophages. The organism can disrupt phagosome maturation, alter antigen processing, and modulate host signaling pathways, allowing survival despite immune pressure. Because of this intracellular niche, antibody induction alone is unlikely to be sufficient; Th1-type responses, macrophage activation, and cytotoxic T-cell activity are also relevant [3,4,5].

This biological setting argues for vaccine candidates that combine antibody recognition with cellular immune activation. It also explains why MAP vaccine development has remained difficult [6].

### 1.2. Limitations of Current MAP Vaccine Strategies

Vaccines currently used or investigated for Johne’s disease are dominated by killed whole-cell preparations and attenuated live formulations. Although such vaccines can reduce shedding and clinical severity, they have limited capacity to prevent persistent infection or maintain durable immune control [6].

Whole-cell vaccines expose the immune system to a wide range of bacterial components and can induce broad responses. That breadth is also a limitation, because shared mycobacterial antigens may interfere with diagnostic assays, and the complexity of these preparations makes it difficult to identify the antigens that drive beneficial immune effects [6].

Single-protein subunit vaccines provide greater antigenic precision, yet this specificity can narrow immune coverage. Because MAP pathogenesis involves invasion, intracellular survival, metabolic adaptation, and immune modulation, targeting one antigen may be insufficient to address the full biology of infection [3,6].

A defined vaccine candidate that combines several immune-relevant targets may therefore be easier to interpret than whole-cell preparations while still avoiding the narrowness of a single-antigen vaccine [6].

### 1.3. Multi-Epitope Vaccines as a Rational Strategy for MAP Vaccine Development

Multi-epitope vaccine design offers one way to combine immune-relevant regions from different antigen sources within a single engineered protein [7].

Unlike whole microorganisms or large antigen mixtures, a multi-epitope construct can be built around predefined immune components. B-cell epitopes are included to support antigen-specific antibody recognition, whereas MHC class I-restricted CTL epitopes and MHC class II-restricted HTL epitopes are intended to engage CD8^+^ and CD4^+^ T-cell responses, respectively [7].

This format is particularly relevant for intracellular pathogens such as MAP, where antibody responses alone are unlikely to provide adequate protection. Control of mycobacterial infection usually involves T-cell responses, IFN-γ-associated macrophage activation, and regulated inflammatory signaling. Combining these epitope classes in one construct may therefore give a broader immune stimulus than a single determinant [3,4,8,9].

At the same time, predicted epitopes cannot be treated as functional epitopes without experimental testing. The expressed construct has to be evaluated after production and administration.

### 1.4. Immunoinformatics-Guided Vaccine Development and Immune Response Characterization

Immunoinformatics and reverse vaccinology can reduce a large pathogen proteome to a smaller group of candidate antigens before laboratory testing. Proteins can be screened for conservation, predicted antigenicity, toxicity, allergenicity, and immune-recognition potential [7,10,11,12].

For MAP, this approach is useful for prioritizing conserved proteins with predicted immune relevance. Structural prediction, docking analysis, and in silico immune simulation can then be used to characterize the resulting construct before experimental evaluation [7].

Most MAP vaccine studies still focus on conventional formulations or individual antigens. Fewer studies link proteome-level screening, multi-epitope design, recombinant expression, immunogenicity testing, and response profiling in the same workflow.

Importantly, immunogenicity is not the same as protection. Challenge experiments are required to measure protective efficacy, whereas antibody and cellular immune assays provide an earlier indication of whether a candidate stimulates relevant immune responses. Transcriptomic profiling of vaccine-responsive cells can further identify pathways associated with early immune activation [7,8].

### 1.5. Objectives of the Present Study

In this study, we used immunoinformatics to design a MAP multi-epitope vaccine candidate and then asked whether the expressed protein showed measurable activity in experimental assays.

Specifically, we selected conserved MAP proteins, assembled predicted B-cell, CTL, and HTL epitopes into one construct, tested the purified protein for antibody and cytokine induction in mice, profiled the transcriptional response of macrophages exposed to the recombinant protein, and evaluated the potential interference of the vaccine candidate with bovine tuberculosis diagnostic antigens.

This was an early immunogenicity and response-profiling study; protection against MAP infection was not tested.

## 2. Materials and Methods

### 2.1. Identification and Screening of MAP Vaccine Antigen Candidates

Protein sequences of *Mycobacterium avium* subsp. *paratuberculosis* (MAP) strain K-10 (ATCC BAA-968; UniProt Taxon ID: 262316) were retrieved from the UniProt database. Candidate proteins were subjected to sequential immunoinformatic screening based on predicted antigenicity, toxicity, allergenicity, and sequence conservation [2,13].

Antigenicity was evaluated using VaxiJen v2.0 with the bacterial model. Potential toxicity and allergenicity were assessed using ToxinPred with default parameters and AllerTOP v2.0 with default parameters, respectively. Proteins showing favorable antigenicity scores and predicted to be non-toxic and non-allergenic were retained for later analyses. The prediction tools, parameter settings, and ranking criteria used for protein and epitope screening are summarized in Table S1. Briefly, candidate proteins were prioritized based on combined consideration of predicted antigenicity, non-toxicity, non-allergenicity, and sequence conservation rather than on a single prediction parameter [10,11,12].

To assess sequence conservation, the selected proteins were compared with homologous proteins from three MAP genome assemblies (GCA_003815795.1, GCA_003957335.1, and GCA_043590545.1). Sequence similarity among homologous proteins was evaluated using NCBI BLASTp (accessed in 2026). Proteins showing relatively high sequence similarity among the examined MAP strains were prioritized as antigen sources for later epitope prediction [2,14].

This screening retained seven MAP proteins as candidate antigen sources: proteasome subunit beta (UniProt ID: Q73YW8), probable regulatory protein (B5LU12), uncharacterized protein (Q73Y48), DUF732 domain-containing protein (Q73Y71), thioredoxin-like fold domain-containing protein (Q73VK8), Mce protein (Q73TV8), and lipoprotein (Q73YG3).

### 2.2. Prediction and Selection of Immune Epitopes

B-cell, MHC class I-restricted cytotoxic T lymphocyte (CTL), and MHC class II-restricted helper T lymphocyte (HTL) epitopes were predicted from the seven selected MAP antigen candidates. Predicted epitopes were further evaluated according to antigenicity, immune-related prediction scores, and predicted solubility before final selection for incorporation into the multi-epitope vaccine construct. Because the intended target hosts of MAP vaccines are ruminants, BoLA alleles were used for MHC-restricted epitope prediction. The mouse immunization experiment was performed as a preliminary assessment of the overall immunogenicity of the recombinant construct, rather than as a direct validation of BoLA-restricted epitope presentation [7,15,16].

#### 2.2.1. B-Cell Epitope Prediction

Linear B-cell epitopes were predicted using the ABCpred server with a peptide length of 16 amino acids. Candidate epitopes were further evaluated for antigenicity using VaxiJen v2.0 and predicted solubility using ProtSol. Final B-cell epitope prioritization was performed using an internally defined weighted score calculated as ABC score × 40% + immunogenicity score × 30% + solubility probability × 30%. The ranking and construct-level assessment led to the selection of five B-cell epitopes with favorable prediction profiles for the final multi-epitope vaccine construct [10,17].

#### 2.2.2. MHC Class I-Restricted CTL Epitope Prediction

MHC class I-binding peptides were predicted using NetMHC v4.0 against a panel of nine bovine leukocyte antigen (BoLA) class I alleles, including BoLA-HD6, BoLA-JSP.1, BoLA-T2c, BoLA-T2b, BoLA-T2a, BoLA-T7, BoLA-D18.4, BoLA-AW10, and BoLA-T5. Candidate CTL epitopes were further evaluated according to their predicted MHC class I-binding characteristics, class I immunogenicity, antigenicity using VaxiJen v2.0, and predicted solubility using ProtSol server. Final CTL epitope prioritization was performed using an internally defined weighted score calculated as class I immunogenicity score × 40% + immunogenicity score × 30% + solubility probability × 30%. The top-ranked CTL candidates were subsequently evaluated at the construct level, considering overall physicochemical properties, immunogenicity score, and predicted three-dimensional structure, before final adjustment of epitope position, inclusion, or exclusion. Using the combined ranking and construct-level evaluation, ten MHC class I-restricted CTL epitopes were retained for vaccine construction [10,15].

#### 2.2.3. MHC Class II-Restricted HTL Epitope Prediction

MHC class II-restricted HTL epitopes were predicted using NetMHCIIpan v4.3 against a panel of seven bovine leukocyte antigen (BoLA) class II alleles, including BoLA-DRB3_0101, BoLA-DRB3_1001, BoLA-DRB3_1101, BoLA-DRB3_1201, BoLA-DRB3_1501, BoLA-DRB3_1601, and BoLA-DRB3_2002. Strong binders were defined using the default NetMHCIIpan criterion of %Rank_EL < 1.0%. Candidate HTL epitopes were additionally evaluated for predicted IFN-γ-inducing potential using the IFNepitope server with default settings, antigenicity using VaxiJen v2.0, and predicted solubility using ProtSol server. Final HTL epitope prioritization was performed using an internally defined weighted score calculated as IFN-γ-inducing score × 40% + immunogenicity score × 30% + solubility probability × 30%. The top-ranked HTL candidates were subsequently evaluated at the construct level, considering overall physicochemical properties, immunogenicity score, and predicted three-dimensional structure, before final adjustment of epitope position, inclusion, or exclusion. This process yielded seven MHC class II-restricted HTL epitopes for inclusion in the final vaccine construct [10,16,18].

### 2.3. Construction of the Multi-Epitope Vaccine Candidate

The selected B-cell, MHC class I-restricted CTL, and MHC class II-restricted HTL epitopes were assembled into a recombinant multi-epitope vaccine construct. The 50S ribosomal protein L7/L12 (UniProt accession: P9WHE3) was incorporated at the N-terminus as an immunostimulatory adjuvant component. The L7/L12 sequence was followed by an EAAAK linker and the short SKKKK motif associated with the Pam3CSK4-based immunostimulatory design. A second EAAAK linker was introduced downstream of the SKKKK motif before the epitope-containing region [7].

**Figure 1:**
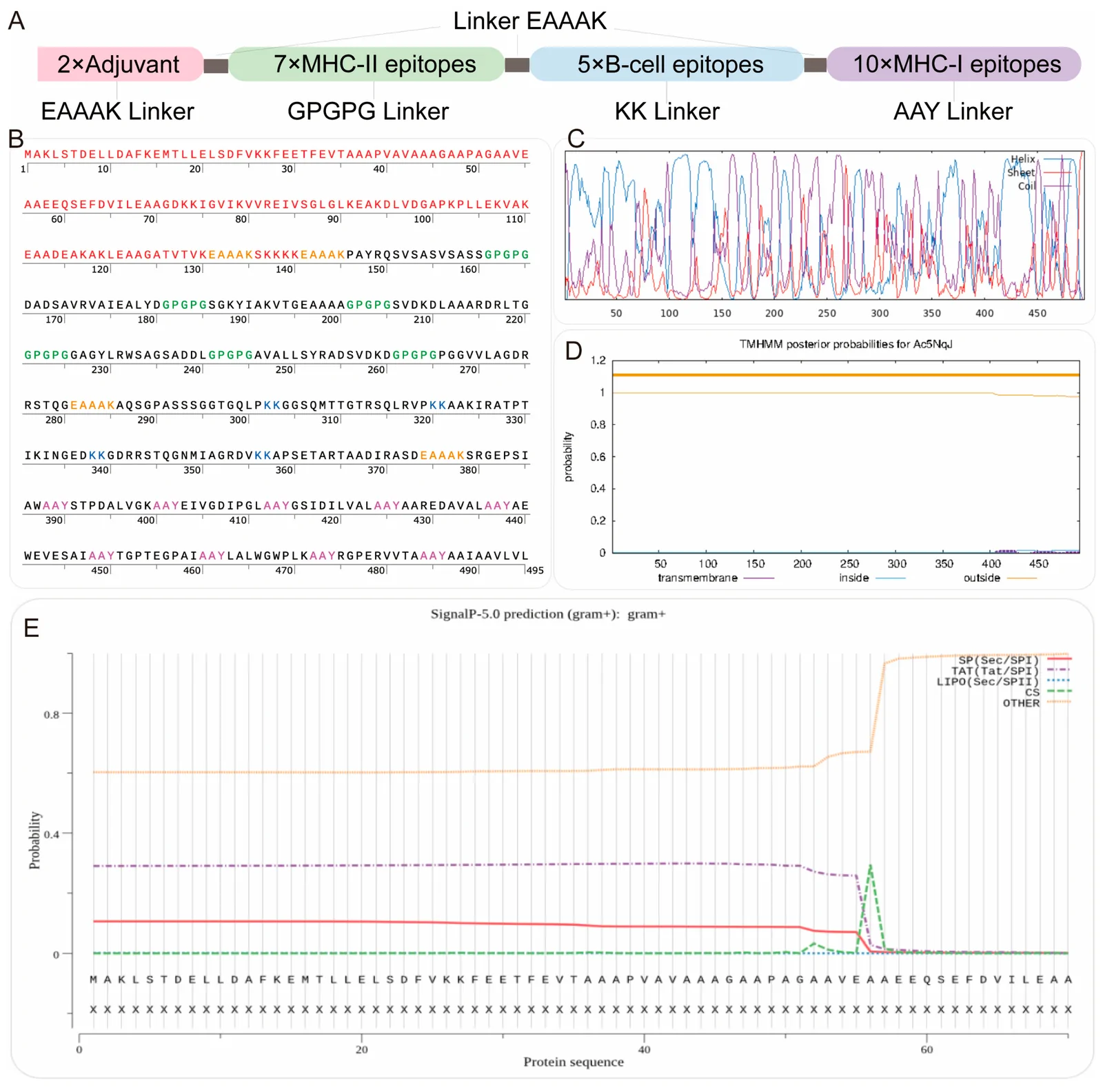
Design and sequence-based characterization of the MAP multi-epitope vaccine candidate. (**A**) Schematic representation of the vaccine construct showing the arrangement of adjuvant sequences, MHC class II epitopes, MHC class I epitopes, and B-cell epitopes connected by different linker sequences. (**B**) Amino acid sequence composition of the designed vaccine construct, with epitope and linker regions highlighted in different colors. (**C**) Predicted secondary structure profile showing the distribution of α-helices, β-strands, coils, and other structural elements. (**D**) Transmembrane topology prediction using TMHMM, indicating the absence of predicted transmembrane regions within the vaccine sequence. (**E**) Signal peptide prediction using SignalP 5.0, showing the probability distribution of different secretion pathway categories along the vaccine sequence.

Individual HTL epitopes were separated using GPGPG linkers. Additional EAAAK and KK linkers were introduced between subsequent epitope-containing regions according to the final construct design. The CTL epitope region was assembled using AAY linkers to facilitate separation of individual MHC class I-restricted epitopes. The final construct contained seven MHC class II-restricted HTL epitopes, five B-cell epitopes, and ten MHC class I-restricted CTL epitopes, arranged in the order HTL, B-cell, and CTL epitope regions as shown in Figure 1A [7].

The complete amino acid sequence of the final multi-epitope construct was as follows:

MAKLSTDELLDAFKEMTLLELSDFVKKFEETFEVTAAAPVAVAAAGAAPAGAAVEAAEEQSEFDVILEAAGDKKIGVIKVVREIVSGLGLKEAKDLVDGAPKPLLEKVAKEAADEAKAKLEAAGATVTVKEAAAKSKKKKEAAAKPAYRQSVSASVSASSGPGPGDADSAVRVAIEALYDGPGPGSGKYIAKVTGEAAAAGPGPGSVDKDLAAARDRLTGGPGPGGAGYLRWSAGSADDLGPGPGAVALLSYRADSVDKDGPGPGPGGVVLAGDRRSTQGEAAAKAQSGPASSSGGTGQLPKKGGSQMTTGTRSQLRVPKKAAKIRATPTIKINGEDKKGDRRSTQGNMIAGRDVKKAPSETARTAADIRASDEAAAKSRGEPSIAWAAYSTPDALVGKAAYEIVGDIPGLAAYGSIDILVALAAYAAREDAVALAAYAEWEVESAIAAYTGPTEGPAIAAYLALWGWPLKAAYRGPERVVTAAAYAAIAAVLVL

The finalized construct was then analyzed for physicochemical properties, predicted structure, molecular docking, and simulated immune responses.

### 2.4. Physicochemical Property Analysis and Structural Prediction

The physicochemical properties of the designed multi-epitope vaccine construct were evaluated using the ExPASy ProtParam server. Molecular weight, theoretical isoelectric point (pI), instability index, aliphatic index, and grand average of hydropathicity (GRAVY) were calculated to characterize the basic physicochemical properties of the construct [19].

Secondary structure was predicted using PSIPRED 4.0 to estimate the distribution of α-helical, β-strand, and coil regions within the vaccine sequence. Potential transmembrane regions were predicted using TMHMM Server v.2.0, and signal peptide prediction was performed using SignalP 5.0 [20,21,22].

The three-dimensional structure of the multi-epitope vaccine candidate was predicted using the AlphaFold Server. The predicted model was then subjected to structural refinement using GalaxyWEB and ModRefiner. Structural quality before and after refinement was evaluated using Ramachandran plot analysis and ProSA-web. The proportion of residues located in favored regions of the Ramachandran plot and the ProSA Z-score were used as indicators of stereochemical quality and overall structural reliability [23,24,25,26].

### 2.5. Molecular Docking Analysis

Molecular docking was performed to evaluate the potential structural interactions between the designed MAP multi-epitope vaccine construct and bovine Toll-like receptors TLR1, TLR2, and TLR6. Because experimentally determined three-dimensional structures of bovine TLR1, TLR2, and TLR6 were not available in the Protein Data Bank (PDB), previously reported and structurally validated bovine TLR models were used as receptor structures. The bovine TLR models were derived from the homology-modeling study of Mansouri et al., in which the extracellular domains of bovine TLR1, TLR2, and TLR6 were constructed using homologous human and murine TLR structures and subsequently evaluated by structural validation and molecular dynamics simulation [9,27].

To evaluate the suitability of the bovine receptor models in greater detail, amino acid sequence identity and similarity between bovine and murine TLR1, TLR2, and TLR6 were evaluated using the Needle pairwise sequence alignment tool provided by NovoPro. Structural superposition between the bovine receptors and their corresponding murine TLR structures was additionally performed using PyMOL 3.1.3.1, and root-mean-square deviation (RMSD) values were calculated to assess overall structural similarity.

Protein-protein docking between the refined multi-epitope vaccine structure and bovine TLR1, TLR2, and TLR6 was performed using the HDOCK server. The refined vaccine structure was used as the ligand, whereas each bovine TLR structure was independently used as the receptor. Docking calculations were performed using the default HDOCK parameters. The top-ranked docking models were selected according to HDOCK docking scores and confidence scores [28].

The selected receptor-vaccine complexes were then analyzed using PDBePISA to characterize the predicted protein-protein interfaces. Interface area, hydrogen bonds, and salt bridges were examined to further describe the predicted interactions between the vaccine construct and each bovine TLR.

Docking and interface analyses were used only to examine predicted structural compatibility between the vaccine construct and the selected innate immune receptors; they were not taken as direct evidence of receptor activation.

### 2.6. In Silico Immune Simulation

In silico immune response simulation was performed using the C-IMMSIM Online server to evaluate the potential immunological responses associated with the designed MAP multi-epitope vaccine construct. The complete amino acid sequence of the finalized vaccine construct was submitted to the server as the antigen input [29].

Simulation outputs included B-cell and plasma-cell responses, helper and cytotoxic T-cell populations, natural killer cells, macrophages, dendritic cells, antigen-specific immunoglobulin production, and cytokine secretion profiles. Simulated antibody responses included IgM- and IgG-associated responses, whereas cytokine outputs included IFN-γ, IL-2, and other immune mediators.

The antigen sequence used for simulation corresponded to the finalized multi-epitope construct described in Section 2.3. MHC class I- and class II-related antigen-presentation outputs generated by the server were included as part of the overall immune simulation analysis.

Changes in immune-cell populations, antigen-specific antibody production, and cytokine levels were examined to characterize the predicted humoral and cellular immune responses associated with the vaccine construct. Default server parameters were used unless otherwise specified.

### 2.7. Sequence Similarity Analysis for DIVA Evaluation of M506070

To evaluate the potential differentiating infected from vaccinated animals (DIVA) compatibility of the designed M506070 multi-epitope vaccine candidate, sequence similarity analyses were performed between M506070 and four commonly used bovine tuberculosis diagnostic antigens, including ESAT-6 (EsxA), CFP-10 (EsxB), MPB70, and MPB83.

The complete amino acid sequence of M506070 was used as the query sequence for pairwise protein alignment against ESAT-6, CFP-10, MPB70, and MPB83 using the NCBI BLASTp online server. Default parameters were applied, and the low-complexity region filter was disabled to allow detection of potential short conserved regions. Sequence identity values and aligned regions were recorded.

To further evaluate potential unexpected similarity with known proteins, the M506070 amino acid sequence was submitted to the UniProtKB protein database for whole-database similarity searching using BLASTp with an E-value threshold of 10. Identified homologous proteins were examined to determine whether they corresponded to known tuberculosis diagnostic antigens or other unrelated mycobacterial proteins.

In addition, all experimentally designed immune epitopes within M506070, including seven MHC class II-restricted HTL epitopes, five B-cell epitopes, and ten MHC class I-restricted CTL epitopes, were individually compared with ESAT-6, CFP-10, MPB70, and MPB83 sequences. Continuous identical amino acid stretches were identified to evaluate potential epitope-level similarity. Potential cross-reactivity was considered unlikely when sequence identity between M506070 and diagnostic antigens was below 30% and no continuous identical amino acid sequence longer than six residues was identified.

### 2.8. Expression and Purification of the Recombinant Vaccine Protein

The codon-optimized gene encoding the finalized MAP multi-epitope vaccine construct was synthesized and cloned into the pET-28a(+) expression vector (Novagen, Madison, WI, USA) containing an N-terminal 6×His tag. The recombinant plasmid was transformed into *Escherichia coli* BL21(DE3) competent cells (TransGen Biotech, Beijing, China) by heat-shock transformation. Positive transformants were selected on LB agar supplemented with kanamycin and confirmed by colony screening.

For recombinant protein expression, a single positive colony was inoculated into LB medium containing kanamycin and cultured overnight at 37 °C. The overnight culture was subsequently transferred into fresh LB medium and grown at 37 °C until the optical density at 600 nm (OD600) reached approximately 0.6. Recombinant protein expression was induced by adding isopropyl β-D-1-thiogalactopyranoside (IPTG) (Sigma-Aldrich, St. Louis, MO, USA) to a final concentration of 1 mM, followed by incubation at 18 °C overnight.

Bacterial cells were harvested by centrifugation and resuspended in phosphate-buffered saline (PBS). Cells were disrupted by ultrasonication at 200 W using 3 s pulses separated by 4 s intervals for 25–30 min. The lysate was centrifuged at 12,000 rpm for 10 min at 4 °C to separate the soluble and insoluble fractions, and recombinant protein expression was evaluated by SDS-PAGE.

The His-tagged recombinant vaccine protein was purified by nickel-affinity chromatography (GE Healthcare, Chicago, IL, USA). Elution fractions from the initial purification were analyzed by SDS-PAGE, and the fraction containing the recombinant target protein was subjected to an additional round of nickel-affinity purification to further enrich the target protein and reduce residual protein contaminants. The resulting purified protein was analyzed by SDS-PAGE, and its identity was further confirmed by Western blotting using an anti-His antibody (Cell Signaling Technology, Danvers, MA, USA).

To minimize potential interference from *E. coli*-derived lipopolysaccharide, endotoxin was removed from the purified recombinant protein using an endotoxin-removal resin (Beyotime Institute of Biotechnology, Shanghai, China) or column. Residual endotoxin levels were quantified using a chromogenic Limulus amebocyte lysate (LAL) assay (Beyotime Institute of Biotechnology, Shanghai, China). The final endotoxin concentration of the purified recombinant protein was confirmed to be below 0.1 EU/mL before its use in subsequent mouse immunization and RAW264.7 (ATCC, Manassas, VA, USA) macrophage-stimulation experiments.

### 2.9. Mouse Immunization, Immunogenicity Evaluation, and Sample Collection

Female BALB/c mice (Laboratory Animal Center of Xinjiang Medical University, Urumqi, China) aged 6 weeks were maintained under specific pathogen-free (SPF) conditions with free access to food and water. All animal experiments were performed in accordance with institutional guidelines for the care and use of laboratory animals and were approved by the Bioethics Committee of Shihezi University (Approval No. A2026-846; approved in March 2026).

To evaluate the immunogenicity of the recombinant MAP multi-epitope vaccine candidate and to perform an exploratory comparison with an MYY-containing formulation, mice were assigned to four experimental groups after body-weight balancing and randomization: PBS control, inactivated MAP, M506070, and M506070-MYY. Each group contained 25 mice. The M506070 group was included to assess the immunogenicity of the recombinant multi-epitope protein without external adjuvant, whereas the M506070-MYY group was used to evaluate the same recombinant protein in an exploratory formulation containing the plant-extract-based nano-adjuvant Mian-Yi-You (MYY; Chinese name: 免易优; Beijing Centrebio Biology Co., Ltd., Beijing, China). The L7/L12 sequence was considered an intrinsic immunostimulatory component of the recombinant design, whereas MYY was evaluated separately as a formulation-level adjuvant. ELISA outcome assessment was performed under blinded conditions.

Mice were immunized subcutaneously at weeks 0, 2, and 4. The PBS control group received 100 μL PBS alone. The inactivated MAP group received 0.1 mg wet weight of heat-inactivated MAP biomass prepared from a MAP strain kindly provided by Xinjiang Academy of Agricultural and Reclamation Sciences. The isolate was cultured in 7H9 medium supplemented with OADC (BD Difco, Franklin Lakes, NJ, USA) for 6–8 weeks, inactivated by incubation at 80 °C for 60 min, and resuspended in PBS. Viability after inactivation was not confirmed. The M506070 group received 50 μg of purified recombinant MAP multi-epitope protein in a total injection volume of 100 μL. The M506070-MYY group received 50 μg of purified recombinant MAP multi-epitope protein formulated with MYY at a 1:1 (*v*/*v*) ratio, with a final injection volume of 100 μL per mouse. MYY was purchased from Beijing Centrebio Biology Co., Ltd. (Beijing, China). The formulation is the subject of a patent application, and its exact active components were not fully defined in the present work.

Blood samples were collected before immunization and at weeks 2, 4, 6, 8, 10, and 12 after immunization for analysis of antigen-specific antibody responses. At each post-immunization time point, serum samples from three mice per group were used for antibody analysis. Serum samples were collected longitudinally from the same mouse cohort, although the same individual mice could not be guaranteed at every time point. Wells with obvious contamination during ELISA were excluded from analysis; no animals or animal samples were excluded from the study. For cellular immune-response analysis, separate subsets of mice were euthanized at weeks 2, 4, and 8 after immunization, and spleens were collected for splenocyte isolation and antigen-restimulation assays as described below.

### 2.10. Determination of Antigen-Specific Antibody Responses

Antigen-specific antibody responses were determined by indirect enzyme-linked immunosorbent assay (ELISA). Ninety-six-well ELISA plates were coated with purified recombinant multi-epitope vaccine protein (M506070) at a concentration of 2 μg/mL in a volume of 100 μL per well and incubated overnight at 4 °C. Plates were washed three times with phosphate-buffered saline containing 0.05% Tween-20 (PBST) and subsequently blocked with 5% bovine serum albumin (BSA) for 1 h at room temperature.

Serum samples collected before immunization and at weeks 2, 4, 6, 8, 10, and 12 after immunization were subjected to two-fold serial dilution from 1:200 to 1:204,800. Diluted serum samples were added to antigen-coated plates at 100 μL per well and incubated for 1 h at room temperature. After three washes with PBST, HRP-conjugated goat anti-mouse IgG (H + L), HRP-conjugated goat anti-mouse IgG1, or HRP-conjugated goat anti-mouse IgG2a (Cell Signaling Technology, Danvers, MA, USA) was added as the secondary antibody, followed by incubation for 1 h at room temperature.

After three additional washes with PBST, bound antibodies were detected using a TMB two-component substrate solution. The reaction was terminated using 1× ELISA stop solution according to the manufacturer’s instructions, and absorbance was measured at 450 nm using a microplate reader (Multiskan FC; Thermo Fisher Scientific, Waltham, MA, USA).

Endpoint antibody titers were defined as the highest serum dilution producing an absorbance value above the predetermined cutoff. Antigen-specific total IgG, IgG1, and IgG2a responses were determined, and the IgG2a/IgG1 ratio was then calculated to assess the relative balance of Th1- and Th2-associated humoral immune responses.

Additional antigen-recognition profiling was performed using week 8 serum samples to evaluate the breadth and specificity of vaccine-induced antibody responses. ELISA plates were coated with purified recombinant M506070 protein, MAP lysate, or purified protein derivative (PPD), respectively. Serum samples from PBS-immunized, inactivated MAP-immunized, M506070-immunized, and M506070-MYY-immunized mice were analyzed. An archived bovine serum sample collected from a tuberculin skin test-positive animal and maintained in our laboratory at Shihezi University was included as a reference positive control for the PPD-reactivity assay. Mouse sera were detected using HRP-conjugated goat anti-mouse IgG, whereas the bovine reference serum was analyzed separately using an HRP-conjugated anti-bovine IgG secondary antibody (Cell Signaling Technology, Danvers, MA, USA). The bovine reference serum was used only as a reference for PPD reactivity and was not included in statistical comparisons among the mouse vaccination groups.

For these assays, sera were serially diluted two-fold starting from 1:100. Endpoint antibody titers were defined as the highest dilution showing an OD450 value above the cutoff determined from PBS-immunized serum controls. Samples that remained below the cutoff at the lowest tested dilution were recorded as below detection limit (<100).

### 2.11. Cellular Immune Response Evaluation

Cell-mediated immune responses were evaluated using splenocytes collected from mice in the PBS, inactivated MAP, M506070, and M506070-MYY groups at weeks 2, 4, and 8 after immunization; at each time point, separate subsets of mice (*n* = 5 per group) were euthanized for spleen collection. For the antigen-specific restimulation assay, splenocytes collected at week 4 from the M506070 group were used (*n* = 3).

Briefly, spleens were mechanically dissociated using sterile cell strainers, and erythrocytes were removed using red blood cell lysis buffer. Isolated splenocytes were washed with sterile culture medium and adjusted to the required cell concentration for subsequent in vitro stimulation assays. Isolated splenocytes were cryopreserved in fetal bovine serum (Thermo Fisher, Waltham, MA, USA) supplemented with 10% dimethyl sulfoxide (DMSO) and stored in liquid nitrogen. Before stimulation, cells were thawed rapidly at 37 °C, washed, and resuspended in fresh culture medium; post-thaw viability was confirmed by trypan blue exclusion.

For antigen-specific stimulation analysis, splenocytes collected at week 4 after immunization were cultured in 96-well plates and stimulated with different antigens, including PBS, purified protein derivative (PPD), MAP lysate, or recombinant M506070 protein. Concanavalin A (ConA, 5 μg/mL) was included as an internal positive control for splenocyte responsiveness and assay performance; ConA-stimulated wells were used for quality control and were not included in the quantitative comparisons. The final concentrations of PPD, MAP lysate, and recombinant M506070 protein were adjusted to 10 μg/mL. PBS was added at an equivalent volume as the negative stimulation control.

Cells were incubated for 48 h at 37 °C in a humidified atmosphere containing 5% CO_2_. After incubation, culture supernatants were collected and stored for cytokine analysis. The concentrations of interferon-gamma (IFN-γ), tumor necrosis factor-alpha (TNF-α), interleukin-2 (IL-2), and interleukin-17 (IL-17) were determined using mouse cytokine ELISA kits (Jiangsu Jingmei Biotechnology Co., Ltd., Yancheng, China) according to the manufacturer’s instructions.

### 2.12. Transcriptomic Sequencing and Analysis

To characterize early transcriptional responses associated with the recombinant MAP multi-epitope vaccine candidate, transcriptomic sequencing was performed using RAW264.7 mouse macrophages stimulated with the recombinant vaccine protein.

RAW264.7 cells were cultured under standard conditions and treated with purified recombinant MAP multi-epitope vaccine protein at a final concentration of 0.1 mg/mL without external adjuvant. Cells maintained under the same culture conditions without vaccine stimulation served as the negative control group (NC). Cells were collected at 6 and 24 h after stimulation, corresponding to the T6h and T24h groups, respectively. Each group included three biological replicates. Total RNA was extracted using TRIzol reagent (Invitrogen, Carlsbad, CA, USA) according to the manufacturer’s instructions.

RNA samples meeting the required concentration and integrity criteria were used for transcriptome library construction. High-throughput RNA sequencing was performed using the BGI DNBSEQ-T7 platform (BGI, Shenzhen, China). Raw sequencing reads were subjected to quality-control procedures, including removal of adaptor sequences and low-quality reads, to generate clean reads for subsequent analysis.

Clean reads were aligned to the *Mus musculus* reference genome (GRCm39) using HISAT2 2.2.1. Transcript assembly and gene-expression quantification were performed using StringTie 3.0.0. Differentially expressed genes (DEGs) among the NC, T6h, and T24h groups were identified using DESeq2 1.46.0. Genes with an adjusted *p* value < 0.05 and |log_2_ fold change| > 1 were considered significantly differentially expressed [30,31,32].

Principal component analysis (PCA) was performed to assess overall transcriptional differences and biological reproducibility among groups. Hierarchical clustering analysis was conducted based on gene-expression profiles. Functional enrichment analyses were performed using Gene Ontology (GO) and Kyoto Encyclopedia of Genes and Genomes (KEGG) pathway analyses to identify biological processes and signaling pathways associated with recombinant-protein-induced transcriptional responses [33,34].

### 2.13. Statistical Analysis

Statistical analyses were performed using GraphPad Prism version 10 (GraphPad Software, Boston, MA, USA). Data are presented as the mean ± standard deviation (SD).

For antigen-specific antibody responses, differences among the PBS, inactivated MAP, M506070, and M506070-MYY groups over time were analyzed using a mixed-effects model, with immunization time and vaccine formulation included as factors and their interaction included in the model, because serum samples were collected longitudinally from the same mouse cohort but the same individual mice could not be guaranteed at every time point. Pairwise comparisons among groups at each time point were then performed using Tukey’s multiple-comparisons test.

For cytokine responses from antigen-stimulated splenocytes, differences among the four vaccine groups and sampling time points were analyzed using ordinary two-way ANOVA, with time and vaccine formulation included as the two factors and their interaction included in the model. Dunnett’s multiple-comparisons test was subsequently used to compare each vaccine group with the PBS control group at the corresponding time point. Comparisons between M506070 and M506070-MYY were used to describe formulation-associated differences in the recombinant-protein response, rather than to establish an independent adjuvant effect of MYY.

For the antigen-recognition ELISA, statistical comparisons among the mouse vaccination groups were performed using one-way ANOVA followed by Tukey’s multiple-comparisons test. For the antigen-specific splenocyte restimulation comparisons, differences among stimulation conditions were analyzed using one-way ANOVA followed by Tukey’s multiple-comparisons test. The archived bovine tuberculosis-positive reference serum was included only as an assay reference and was not included in the statistical analysis. A *p*-value < 0.05 was considered statistically significant. For graphical presentation, statistical significance was indicated as follows: *p* < 0.05 (*), *p* < 0.01 (**), *p* < 0.001 (***), and *p* < 0.0001 (****); ns, not significant.

## 3. Results

### 3.1. Identification of Conserved and Immunogenic MAP Antigen Candidates

Potential antigen sources for construct design were identified by screening the MAP proteome with an immunoinformatics workflow. Candidate proteins were assessed for conservation and predicted immunological suitability, including antigenicity, immunogenicity, toxicity, and allergenicity.

This screening retained seven proteins as antigen sources: proteasome subunit beta, probable regulatory protein, uncharacterized protein, DUF732 domain-containing protein, thioredoxin-like fold domain-containing protein, Mce protein, and lipoprotein. These proteins met the predefined prediction criteria and were classified as non-toxic and non-allergenic (Table S2).

The predicted immunogenicity scores of the selected proteins ranged from 1.0194 to 1.0356. Among the seven candidates, the Mce protein exhibited the highest predicted immunogenicity score (1.0356), followed by the thioredoxin-like fold domain-containing protein (1.0328), whereas the DUF732 domain-containing protein showed the lowest score (1.0194).

BLASTp-based sequence comparison among different MAP strains indicated a high degree of conservation for several selected proteins, whereas some candidates showed comparatively lower similarity (Table S3). The proteasome subunit beta and thioredoxin-like fold domain-containing protein showed 100% sequence similarity among the examined strains. The probable regulatory protein showed 98.1–99.5% similarity, and the DUF732 domain-containing protein showed 84.3% similarity across the examined strains. The uncharacterized protein showed 99.3–100% similarity, and the Mce protein showed 91.4–91.8% similarity. The lipoprotein exhibited comparatively lower sequence conservation, with similarity values ranging from 74.1% to 78.9%.

These prediction and conservation data guided the choice of antigen sources for the multi-epitope design.

### 3.2. Identification of Immune Epitopes and Construction of the MAP Multi-Epitope Vaccine Candidate

B-cell, MHC class I-restricted CTL, and MHC class II-restricted HTL epitopes were then screened from the selected antigen candidates. Because MHC-restricted epitopes were predicted against bovine BoLA alleles, they should be regarded as target-host-oriented computational candidates rather than experimentally confirmed epitopes.

#### 3.2.1. Identification of B-Cell Epitopes

Linear B-cell epitope prediction identified multiple peptide regions with favorable predicted antigenic properties. Candidate epitopes were further evaluated according to prediction score, antigenicity, and solubility. Based on the integrated selection criteria, five B-cell epitopes were selected for incorporation into the final multi-epitope vaccine construct, and their sequences are highlighted within the complete construct sequence in Figure 1B and summarized in Table S4.

#### 3.2.2. Identification of MHC Class I-Restricted CTL Epitopes

MHC class I-restricted CTL epitopes were screened from the selected MAP antigen candidates. Candidate peptides were evaluated according to predicted MHC class I-binding characteristics, immunogenicity, antigenicity, and solubility. Based on the integrated selection criteria, ten CTL epitopes were selected for incorporation into the final construct, and their sequences are highlighted within the complete construct sequence in Figure 1B and summarized in Table S4.

#### 3.2.3. Identification of MHC Class II-Restricted HTL Epitopes

MHC class II-restricted HTL epitopes were screened based on predicted MHC class II-binding characteristics, IFN-γ-inducing potential, antigenicity, and solubility. Following integrated evaluation, seven HTL epitopes were selected for vaccine construction, and their sequences are highlighted within the complete construct sequence in Figure 1B and summarized in Table S4.

#### 3.2.4. Construction and Sequence-Based Characterization of the Multi-Epitope Vaccine Candidate

The selected seven HTL epitopes, five B-cell epitopes, and ten CTL epitopes were assembled with the immunostimulatory component and appropriate linker sequences to generate the final recombinant multi-epitope vaccine construct. The overall organization of the construct is shown in Figure 1A,B.

Different linker sequences were incorporated to separate individual functional regions and epitope groups within the construct. Sequence-based characterization of the resulting vaccine candidate included secondary-structure prediction, transmembrane-region analysis, and signal-peptide prediction (Figure 1C–E). No predicted transmembrane region was identified within the vaccine sequence.

These analyses defined the construct that was carried forward to structural modeling and experimental testing.

### 3.3. Sequence Similarity Analysis Supports Potential DIVA Compatibility

Sequence similarity analysis was performed to assess the potential similarity between M506070 and four commonly used bovine tuberculosis diagnostic antigens. No sequence identity above 30% was detected between M506070 and ESAT-6, CFP-10, MPB70, or MPB83. The longest continuous identical amino acid stretches across the full sequences were 4, 3, 6, and 5 residues, respectively, and none of the corresponding epitope regions contained a continuous identical stretch longer than four residues. Based on the predefined criteria, no substantial sequence similarity or predicted epitope-level cross-reactivity was identified between M506070 and these diagnostic antigens (Table 1).

To further assess potential unexpected similarity across the protein database, M506070 was searched against the UniProtKB database using BLASTp with an E-value threshold of 10. A total of 250 similar sequences were identified, all corresponding to ribosomal protein L7/L12 from different mycobacterial species, consistent with the L7/L12-derived immunostimulatory module incorporated into the construct. No homologous sequences corresponding to ESAT-6, CFP-10, MPB70, or MPB83, or unexpected homology within the predicted epitope regions, were identified. Together, these results indicated limited sequence similarity between M506070 and the major bovine tuberculosis diagnostic antigens and supported the potential DIVA-compatible characteristics of the construct.

### 3.4. Structural Modeling and Molecular Docking Analysis

**Figure 2:**
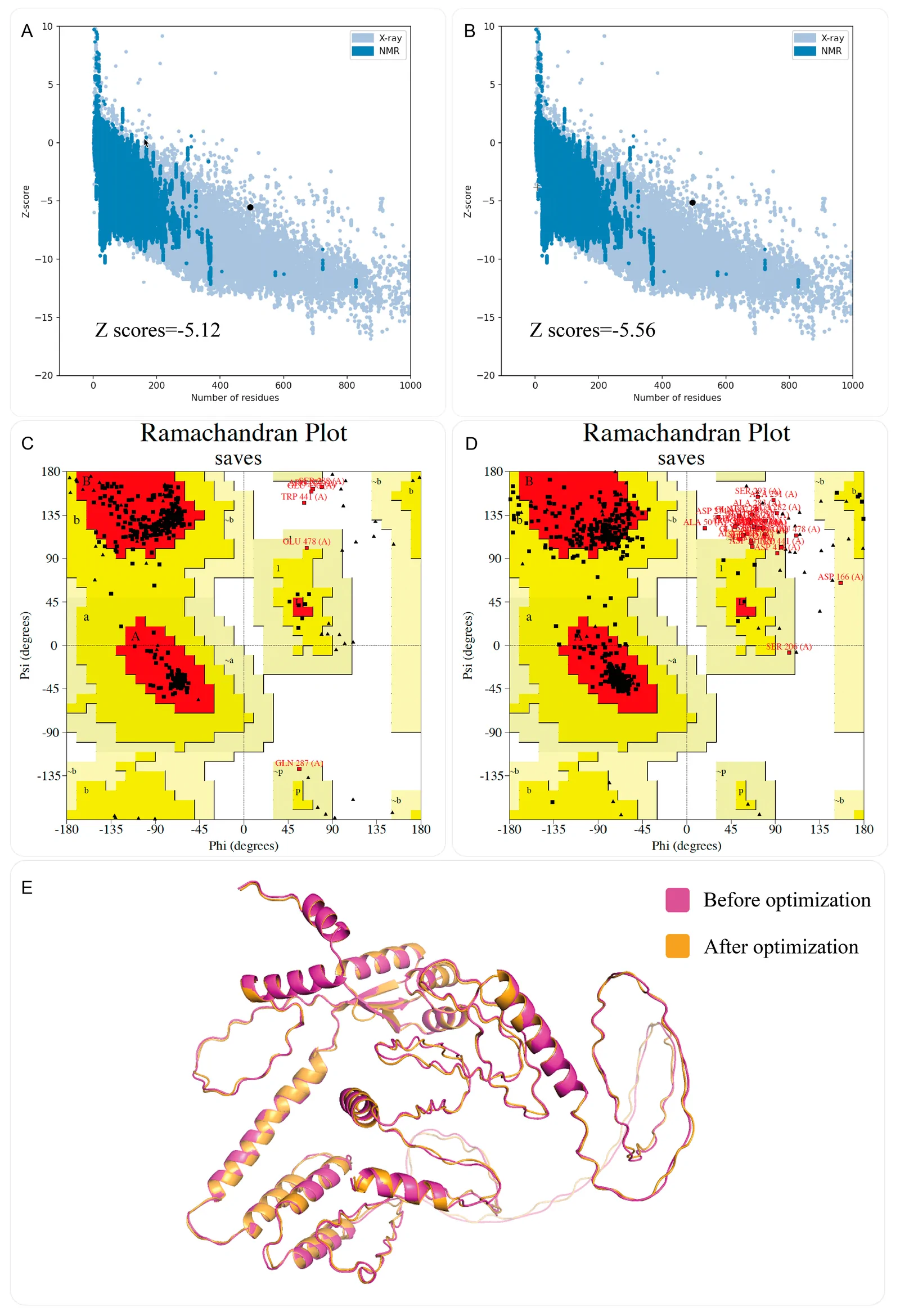
Structural prediction, refinement, and validation of the MAP multi-epitope vaccine candidate. (**A**) Three-dimensional structure prediction of the initial multi-epitope vaccine model. (**B**) Three-dimensional structure of the refined vaccine model after structural optimization. (**C**) Ramachandran plot analysis of the initial vaccine model showing residue distribution and stereochemical quality. (**D**) Ramachandran plot analysis of the refined vaccine model demonstrating improved residue distribution within favored regions. (**E**) Structural comparison between the initial and optimized vaccine models. Structural refinement improved the predicted quality of the vaccine structure, with the ProSA Z-score changing from −5.12 to −5.56 and the proportion of residues in the most favored Ramachandran regions increasing from 79.6% to 94.8%.

The three-dimensional structure of the MAP multi-epitope vaccine candidate was predicted and subsequently refined. Structural refinement improved the overall quality of the predicted model (Figure 2). The proportion of residues located within the most favored regions of the Ramachandran plot increased from 79.6% before refinement to 94.8% after refinement. In parallel, the ProSA Z-score changed from −5.12 to −5.56, indicating improved predicted structural quality following refinement.

**Figure 3:**
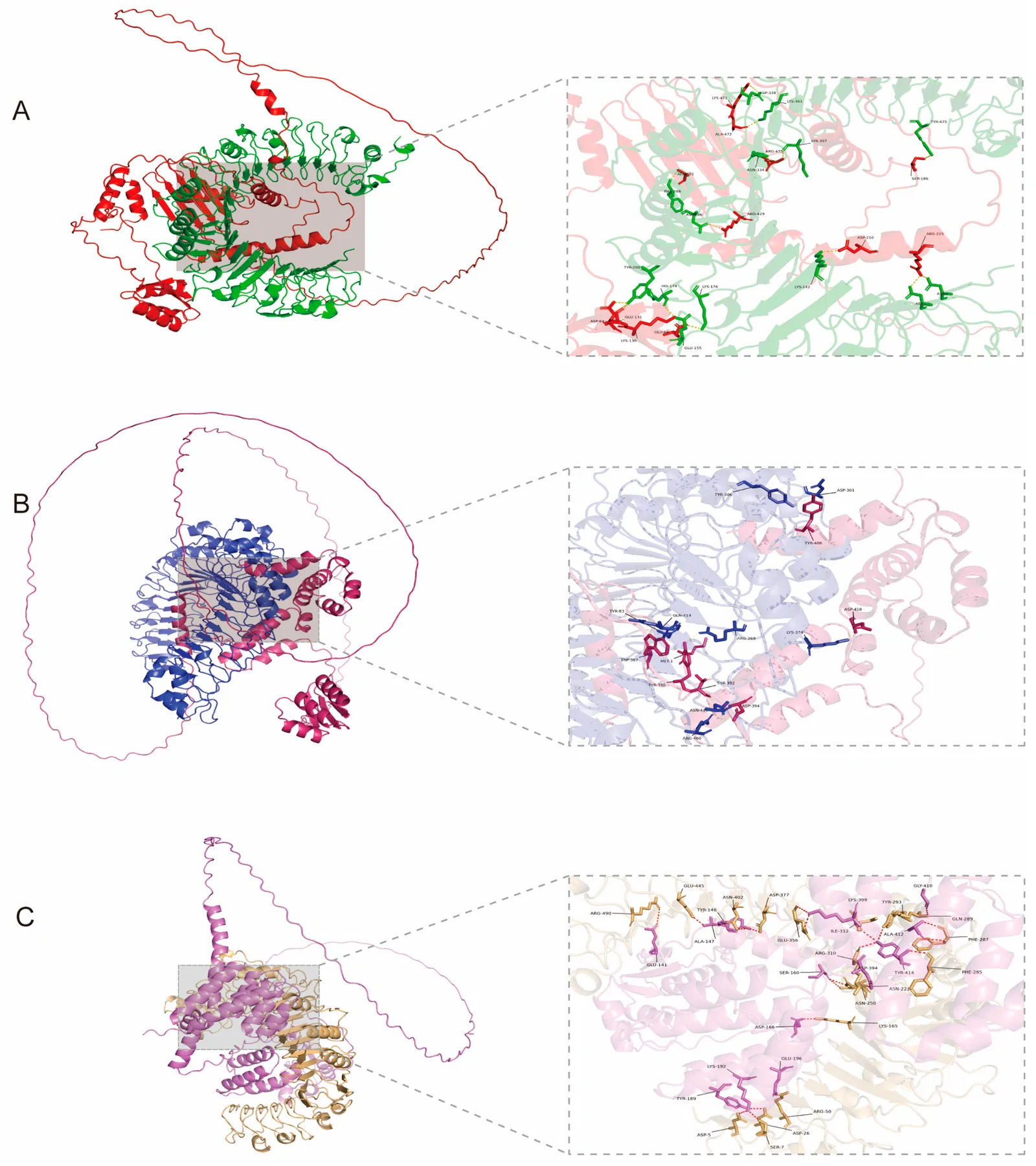
Molecular docking analysis of the MAP multi-epitope vaccine candidate with bovine innate immune receptors. (**A**) Docking model showing the interaction between the vaccine construct and bovine TLR1. (**B**) Docking model showing the interaction between the vaccine construct and bovine TLR2. (**C**) Docking model showing the interaction between the vaccine construct and bovine TLR6. The docking analysis predicted favorable structural interactions between the vaccine construct and the three bovine TLR models. Quantitative analysis showed negative HDOCK scores and multiple predicted intermolecular contacts, including hydrogen bonds and salt bridges. In the docking models, M506070 and receptor molecules are shown in different colors: TLR1 is shown in green and M506070 is shown in red in panel (**A**); TLR2 is shown in blue and M506070 is shown in pink in panel (**B**); and TLR6 is shown in yellow and M506070 is shown in pink in panel (**C**).

The refined vaccine structure was then subjected to molecular docking with bovine TLR1, TLR2, and TLR6 to evaluate potential structural compatibility with these innate immune receptors (Figure 3).

Top-ranked docking models were obtained for all three receptor-vaccine combinations. The TLR6-vaccine complex showed the most favorable HDOCK score (−256.89), followed by TLR2 (−231.93) and TLR1 (−226.30).

Protein-protein interface analysis further characterized the predicted receptor-vaccine interactions. The TLR1-vaccine complex exhibited an interface area of 2824.43 Å^2^ and contained 20 predicted hydrogen bonds and nine salt bridges. The TLR2-vaccine complex had an interface area of 2771.82 Å^2^, with 13 predicted hydrogen bonds and four salt bridges. The TLR6-vaccine complex exhibited an interface area of 2667.56 Å^2^ and contained 24 predicted hydrogen bonds and eight salt bridges.

The docking results were compatible with predicted interactions between the construct and the modeled bovine TLR1, TLR2, and TLR6 receptors, with TLR6 producing the most favorable HDOCK score. These results remain computational interaction predictions and should not be interpreted as direct evidence of receptor binding or activation.

### 3.5. In Silico Immune Simulation Predicts Broad Immune Response Potential

**Figure 4:**
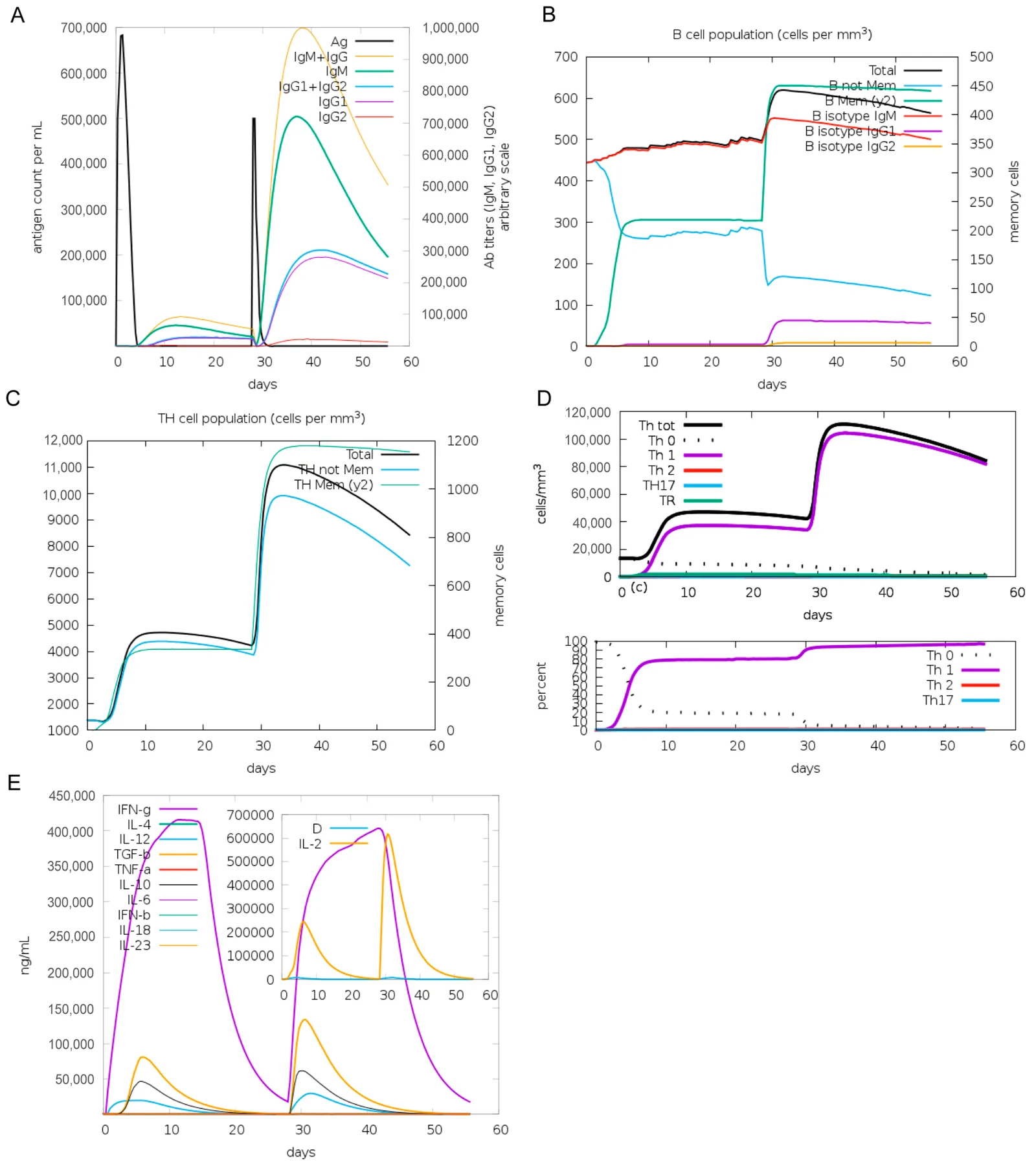
In silico immune simulation of the MAP multi-epitope vaccine candidate. (**A**) Predicted antigen-specific antibody responses following sequential antigen exposure. (**B**) Simulated B-cell population dynamics and predicted memory B-cell development. (**C**) Predicted helper T-cell response. (**D**) Predicted cytotoxic T-cell response. (**E**) Simulated cytokine profile, including IFN-γ, IL-2, and other immune-associated mediators. In summary, the C-IMMSIM analysis predicted coordinated humoral and cellular immune response potential associated with the designed vaccine construct. The simulated response represented by the red curve remained at the baseline level throughout the simulation and therefore overlaps the x-axis; overlapping traces at zero are not visually resolved.

The designed multi-epitope vaccine construct was also examined by in silico immune simulation (Figure 4).

The simulated response showed progressive increases in antigen-specific immunoglobulin production following sequential antigen exposure, including IgM- and IgG-associated responses (Figure 4A). Changes in simulated B-cell populations were also observed, together with the predicted development of memory B-cell populations (Figure 4B).

In addition to humoral responses, the simulation predicted changes in both helper T-cell and cytotoxic T-cell populations following antigen exposure (Figure 4C,D). Cytokine outputs further showed predicted increases in immune-associated mediators, including IFN-γ and IL-2, together with additional inflammatory mediators represented in the simulation (Figure 4E).

The C-IMMSIM output predicted engagement of both humoral and cellular immune components. This result was treated as a computational screen rather than evidence of vaccine-induced immunity in vivo.

### 3.6. Expression and Purification of the Recombinant MAP Multi-Epitope Vaccine Protein

To produce the designed construct, the codon-optimized multi-epitope gene was cloned into pET-28a(+) and expressed in *Escherichia coli* BL21(DE3). Expression was induced with 1 mM IPTG at an OD600 of approximately 0.6, and recombinant protein production was assessed by SDS-PAGE.

**Figure 5:**
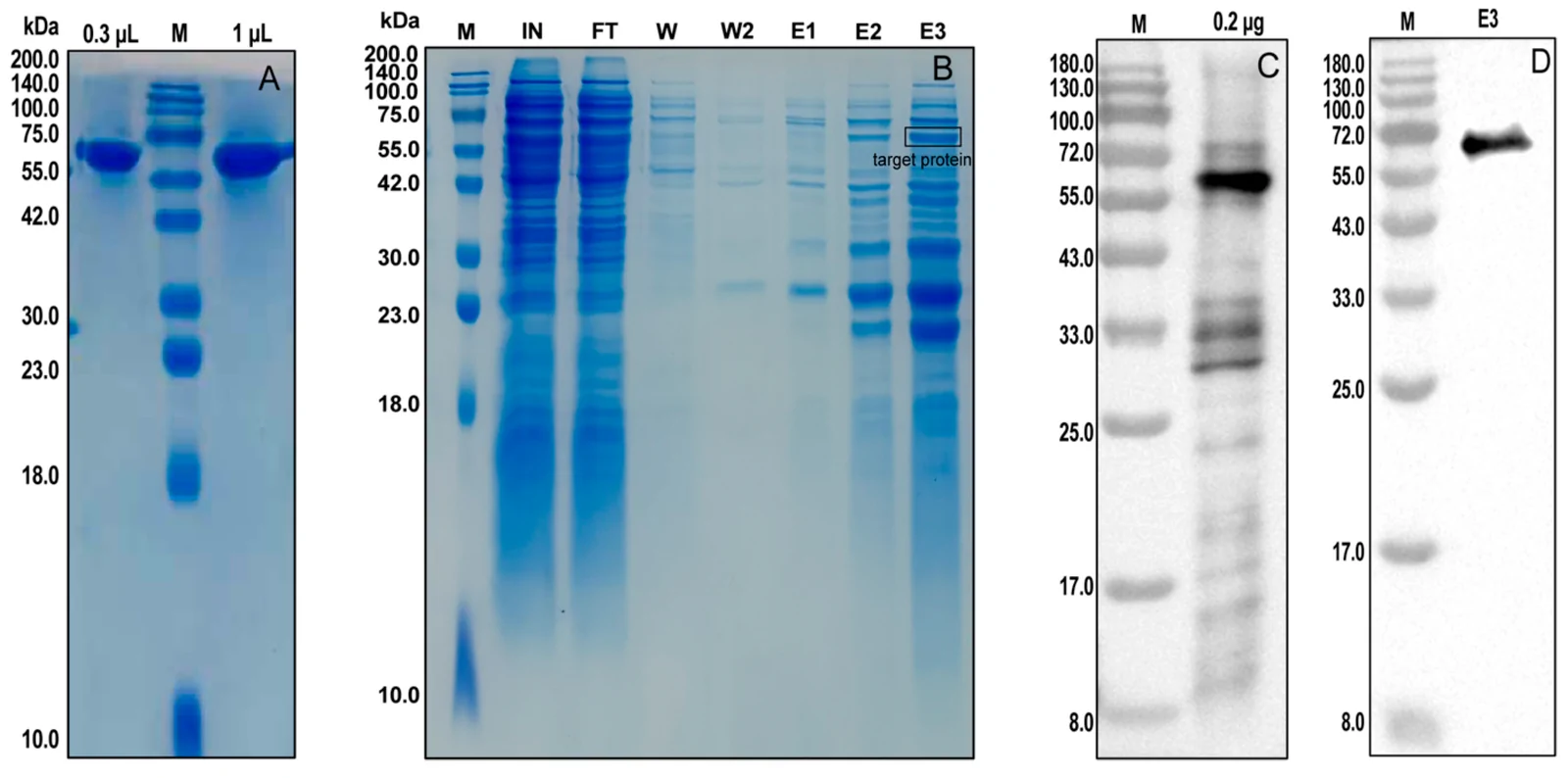
Expression, purification, and identification of the recombinant MAP multi-epitope vaccine protein. (**A**) SDS-PAGE analysis of the purified recombinant protein obtained after additional nickel-affinity purification, shown at two loading volumes (0.3 μL and 1 μL). (**B**) SDS-PAGE analysis of the initial nickel-affinity purification. M, protein marker; IN, induced bacterial lysate; FT, flow-through fraction; W and W2, wash fractions; E1–E3, elution fractions. The recombinant target protein at approximately 55–60 kDa is indicated in the E3 fraction. (**C**) SDS-PAGE analysis of the purified recombinant protein obtained after additional nickel-affinity purification (0.2 μg). (**D**) Western blot analysis of the purified recombinant protein using an anti-His antibody, confirming the identity of the His-tagged recombinant target protein at the expected molecular weight.

The initial nickel-affinity purification generated several elution fractions (E1–E3). SDS-PAGE analysis showed that the E3 fraction contained a prominent band at approximately 55–60 kDa, corresponding to the expected molecular weight of the recombinant protein, together with residual protein bands (Figure 5B).

To further enrich the recombinant protein and reduce residual protein contaminants, the target-protein-containing eluate was subjected to an additional round of nickel-affinity purification. The resulting purified protein showed a prominent band at an apparent molecular weight of approximately 55–60 kDa (Figure 5A,C), close to the predicted molecular weight of 54.4 kDa.

Western blotting of the resulting purified protein using an anti-His antibody detected a single immunoreactive band at the expected molecular weight (Figure 5D), further confirming the identity of the His-tagged recombinant target protein. The purified protein was subsequently used for mouse immunization and macrophage stimulation experiments.

The final purified protein preparation contained <0.1 EU/mL endotoxin and was used for subsequent immunization and macrophage-stimulation experiments.

### 3.7. The MAP Multi-Epitope Vaccine Induces Sustained Antigen-Specific Humoral Immune Responses

**Figure 6:**
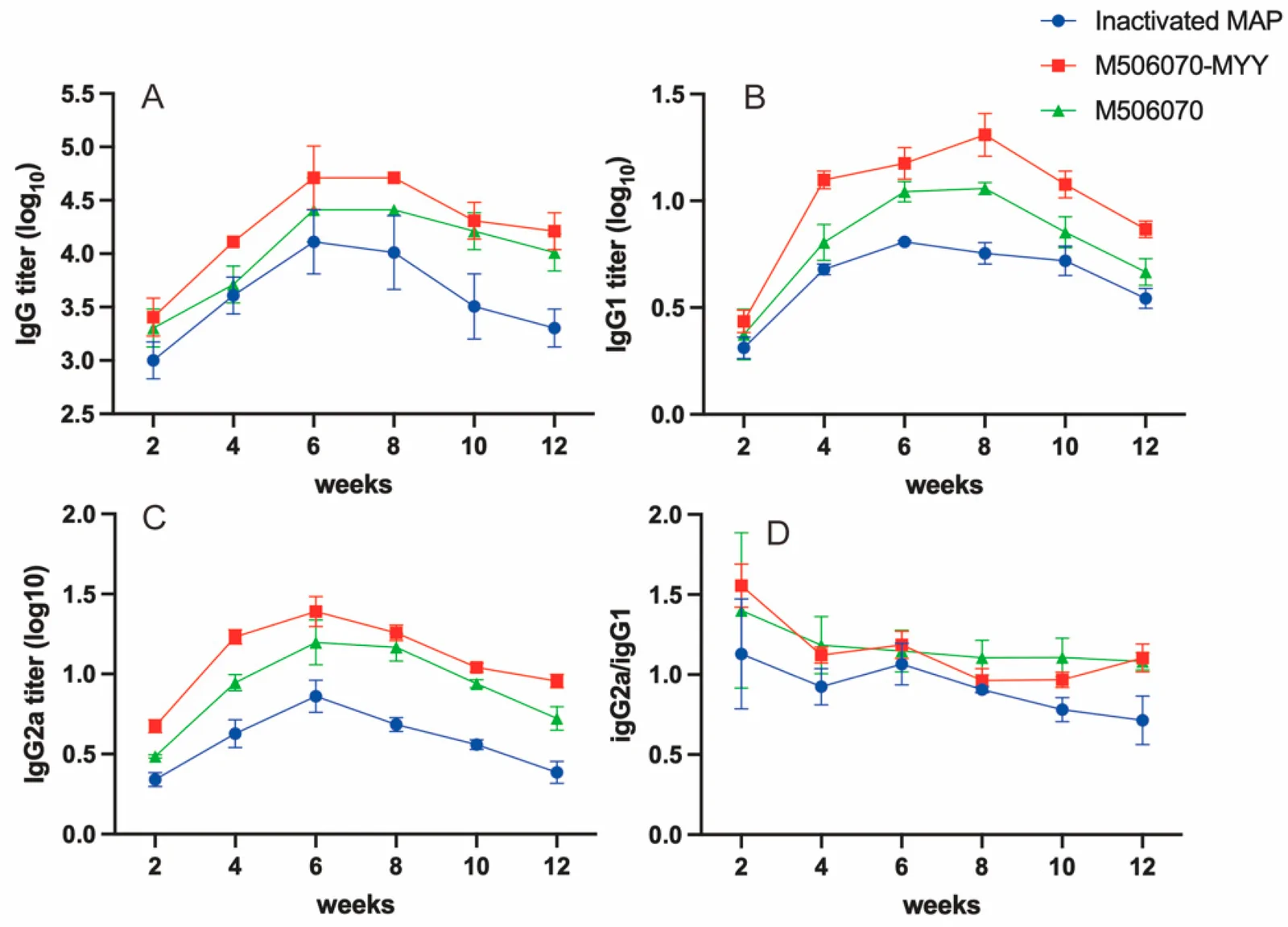
Antigen-specific humoral immune responses induced by different MAP vaccine groups. (**A**) Antigen-specific total IgG responses, (**B**) antigen-specific IgG1 responses, (**C**) antigen-specific IgG2a responses, and (**D**) IgG2a/IgG1 ratios were determined at weeks 2, 4, 6, 8, 10, and 12 after immunization in mice receiving inactivated MAP, M506070, or M506070-MYY (PBS-immunized mice served as negative controls). Antigen-specific antibody responses were measured by indirect ELISA. Data are presented as mean ± SD (*n* = 3 per group at each time point). Statistical significance was assessed using a mixed-effects model followed by Tukey’s multiple-comparisons test, with pairwise comparisons among groups performed at each time point. *p* < 0.05 was considered statistically significant.

To evaluate vaccine-induced humoral immunity, antigen-specific total IgG, IgG1, and IgG2a responses were monitored at multiple time points following immunization (Figure 6).

Antigen-specific total IgG responses increased after immunization and reached relatively high levels at approximately weeks 6–8 (Figure 6A). The MYY-free M506070 group showed detectable antigen-specific IgG responses, indicating that the recombinant protein was immunogenic without an external adjuvant. The M506070-MYY group showed numerically higher total IgG levels than the M506070 and inactivated MAP groups during parts of the later observation period, although these differences did not reach statistical significance at the evaluated time points; this comparison should therefore be interpreted as formulation-associated and exploratory rather than as evidence of an MYY-specific adjuvant effect.

Analysis of IgG subclasses showed that antigen-specific IgG1 responses increased after immunization and reached their highest levels at approximately weeks 6–8 (Figure 6B). The M506070-MYY group showed numerically higher IgG1 levels than the MYY-free M506070 group and the inactivated MAP group at several post-immunization time points; however, these differences were not statistically significant, and the absence of an MYY-only group limits attribution of the observed pattern specifically to MYY.

A pronounced group-dependent response was also observed for antigen-specific IgG2a (Figure 6C). The M506070-MYY group exhibited numerically higher IgG2a levels than the M506070 and inactivated MAP groups at multiple evaluated time points without reaching statistical significance; this pattern was therefore treated as a formulation-associated observation rather than independent evidence of MYY-specific activity.

The IgG2a/IgG1 ratio remained generally close to 1 across the evaluated groups and time points (Figure 6D), indicating that both IgG1- and IgG2a-associated antibody responses were induced without a sustained predominance of either subclass.

Thus, antibody responses varied over time after immunization and showed formulation-associated differences. The recombinant MAP multi-epitope protein was immunogenic without an external adjuvant, and the exploratory MYY-containing formulation was associated with numerically higher antigen-specific humoral readouts involving both IgG1 and IgG2a, although these differences did not reach statistical significance.

### 3.8. Antigen Recognition Profile of M506070-Induced Antibodies

**Figure 7:**
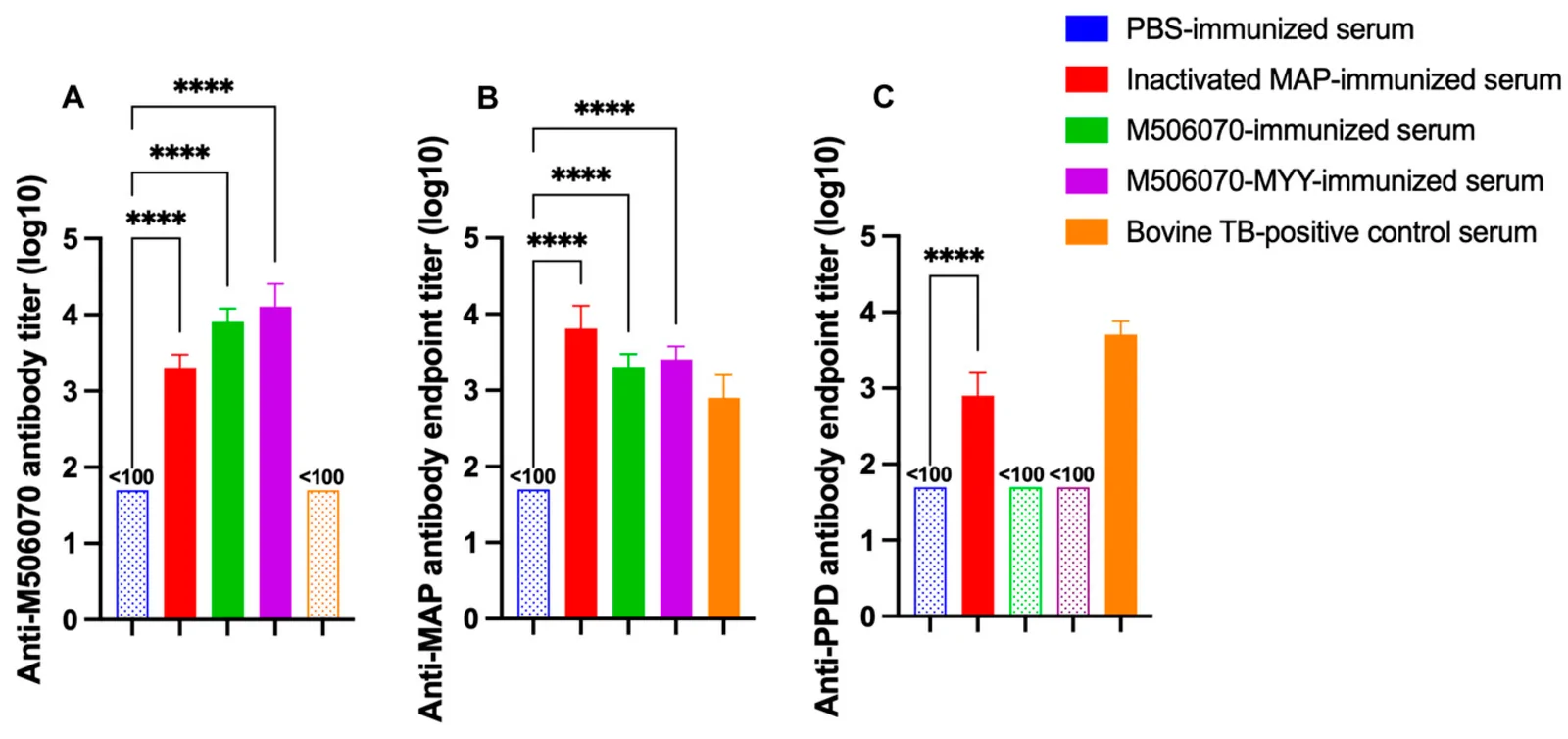
Antigen recognition profiles of serum antibodies induced by different MAP vaccine formulations. (**A**) Endpoint titers of M506070-specific antibodies determined by ELISA using recombinant M506070 protein as the coating antigen. (**B**) Endpoint titers of MAP-associated antibodies determined by ELISA using MAP lysate as the coating antigen. (**C**) Endpoint titers of PPD-specific antibodies determined by ELISA using purified protein derivative (PPD) as the coating antigen. Patterned bars indicate antibody titers below the detection limit (<100), whereas solid bars indicate detectable antibody titers. Serum samples were collected at week 8 after immunization from mice receiving PBS, inactivated MAP, M506070, or M506070-MYY formulations. An archived bovine serum sample collected from a tuberculin skin test-positive animal was included as a reference control for PPD reactivity and was analyzed separately using an anti-bovine IgG secondary antibody. The bovine reference serum was not included in statistical comparisons among the mouse vaccination groups. Endpoint antibody titers were determined as the highest serum dilution showing an OD450 value above the predefined cutoff. Data are presented as mean ± SD (*n* = 3 mice). Statistical significance was assessed using one-way ANOVA followed by Tukey’s multiple-comparisons test. *p* < 0.0001 (****).

To further characterize the specificity of vaccine-induced antibody responses, week 8 serum samples were evaluated against different coating antigens, including recombinant M506070 protein, MAP lysate, and PPD (Figure 7).

When M506070 was used as the coating antigen, both M506070-immunized and M506070-MYY-immunized mice showed strong antigen-specific antibody recognition, whereas PBS-immunized mice remained below the detection threshold. The archived serum from the tuberculin skin test-positive animal showed little or no reactivity to recombinant M506070. Inactivated MAP-immunized mice also showed detectable recognition of M506070, consistent with exposure to MAP-derived antigens.

When MAP lysate was used as the coating antigen, inactivated MAP-immunized mice exhibited broad antibody recognition. Importantly, sera from M506070 and M506070-MYY groups also recognized MAP lysate, indicating that antibodies induced by the recombinant multi-epitope vaccine could recognize components present in MAP lysate.

In contrast, PPD showed strong reactivity with the archived serum from the tuberculin skin test-positive animal and moderate recognition by sera from inactivated MAP-immunized mice, whereas sera from the M506070 and M506070-MYY groups showed limited reactivity.

These results indicate that M506070 vaccination induced MAP-associated antibody responses while maintaining limited recognition of PPD, supporting the potential diagnostic compatibility of this multi-epitope vaccine strategy.

### 3.9. M506070 Vaccination Induces Antigen-Specific Cellular Responses Following Restimulation with Different Antigens

**Figure 8:**
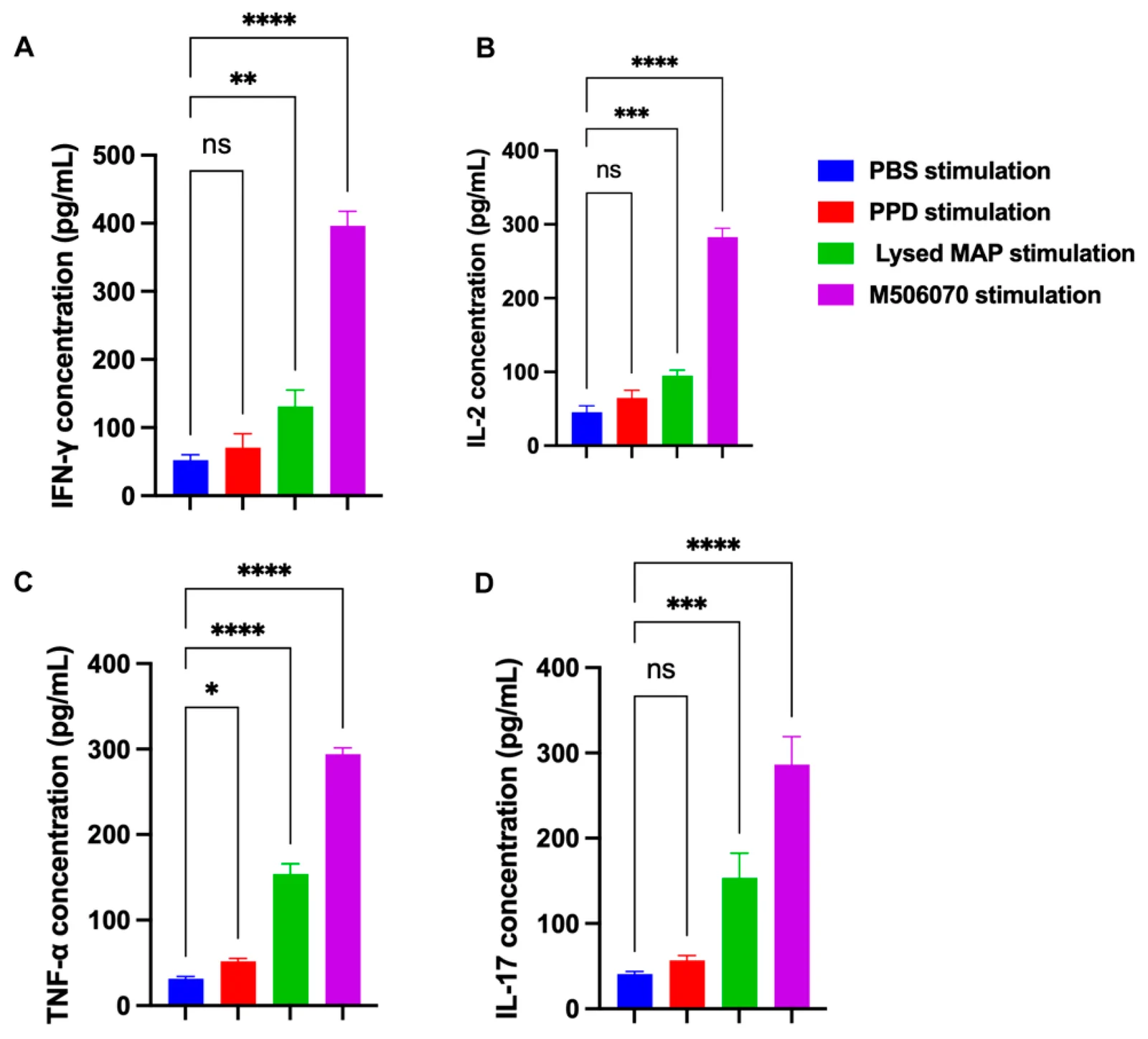
Antigen-specific cytokine responses of splenocytes from M506070-immunized mice following different restimulation conditions. (**A**) IFN-γ, (**B**) IL-2, (**C**) TNF-α, and (**D**) IL-17 concentrations in splenocyte culture supernatants following antigen-specific stimulation. Splenocytes were collected at week 4 after immunization from mice in the M506070 group and stimulated in vitro with PBS, purified protein derivative (PPD), MAP lysate, or recombinant M506070 protein for 48 h. Cytokine concentrations were determined using mouse cytokine ELISA kits according to the manufacturer’s instructions. Data for the mouse vaccination groups are presented as mean ± SD (*n* = 3 mice per group). Statistical significance was assessed using one-way ANOVA followed by Tukey’s multiple-comparisons test. *p* < 0.05 (*), *p* < 0.01 (**), *p* < 0.001 (***), and *p* < 0.0001 (****); ns, not significant.

To further evaluate the cellular immune responses induced by the MAP multi-epitope vaccine, splenocytes collected at week 4 after immunization were restimulated with different antigen preparations, including PBS, PPD, MAP lysate, and recombinant M506070 protein. Cytokine secretion in culture supernatants was subsequently measured by ELISA (Figure 8).

In contrast, PBS stimulation resulted in low baseline secretion of IFN-γ, TNF-α, IL-2, and IL-17.

Following antigen-specific stimulation, recombinant M506070 induced the strongest cytokine responses among all tested antigens. Splenocytes from M506070-immunized mice showed increased secretion of IFN-γ, TNF-α, IL-2, and IL-17 compared with PBS stimulation, indicating effective antigen-specific recall responses.

MAP lysate stimulation also induced cytokine production, although the levels were lower than those induced by recombinant M506070 stimulation. This suggested that the immune response generated against the designed multi-epitope antigen could recognize components present in MAP lysate.

In comparison, PPD stimulation induced limited cytokine secretion in M506070-immunized mice, with responses remaining close to PBS controls. This pattern was consistent with the limited PPD recognition observed in the antibody-based ELISA analysis.

Overall, these results demonstrated that M506070 vaccination elicited antigen-specific cellular immune responses characterized by enhanced production of Th1-associated cytokines (IFN-γ, TNF-α, and IL-2) and the Th17-associated cytokine IL-17, while maintaining limited responsiveness toward the conventional tuberculosis diagnostic antigen PPD.

### 3.10. Kinetics of Antigen-Specific Cellular Responses Following MAP Multi-Epitope Vaccination

**Figure 9:**
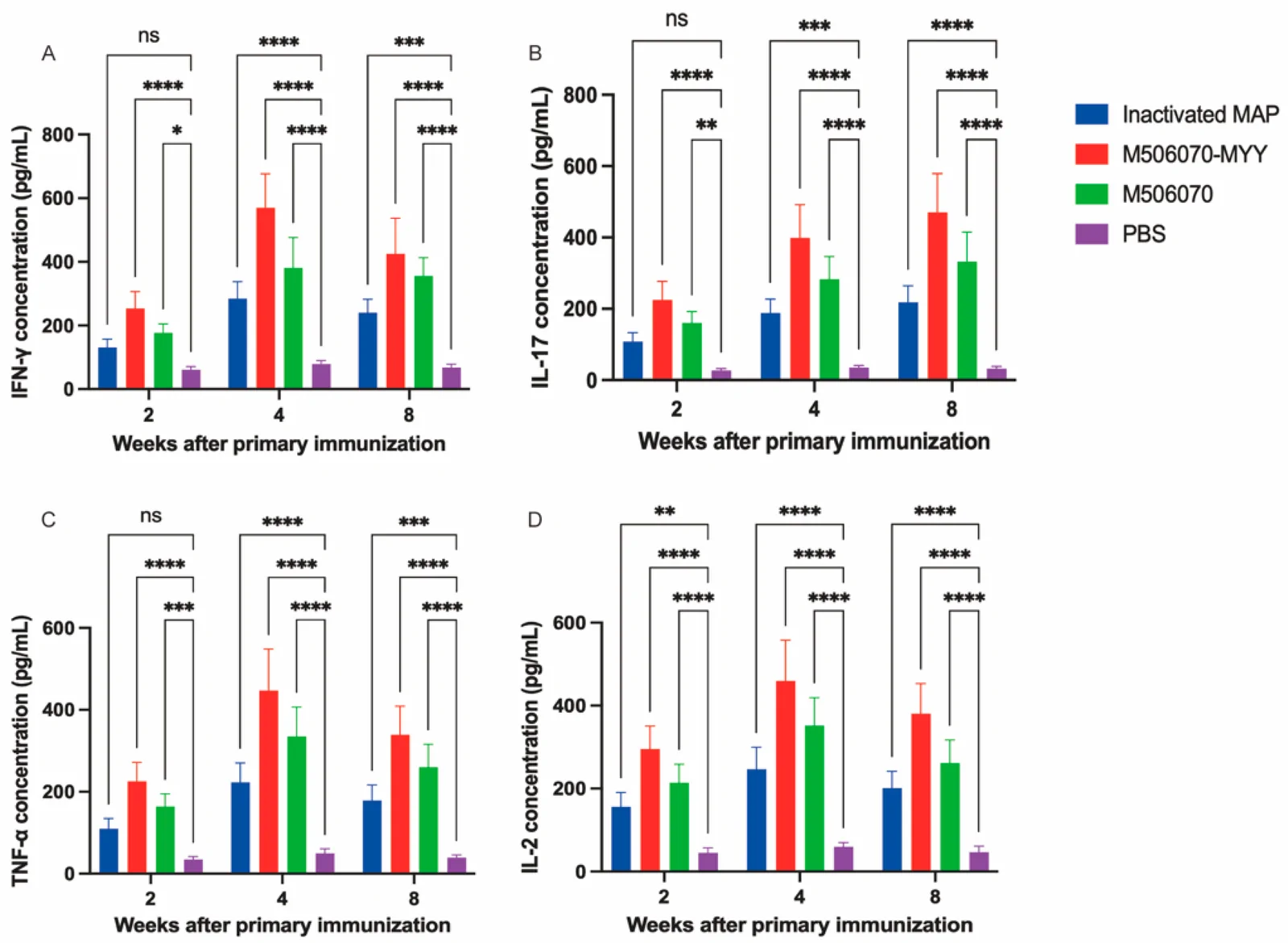
Cytokine responses of antigen-stimulated splenocytes following immunization with different MAP vaccine groups. Splenocytes were isolated from separate subsets of mice at weeks 2, 4, and 8 after immunization and restimulated ex vivo with the recombinant MAP multi-epitope vaccine protein for 48 h. Cytokine concentrations in the culture supernatants were determined by ELISA. (**A**) IFN-γ, (**B**) IL-17, (**C**) TNF-α, and (**D**) IL-2 concentrations in splenocyte culture supernatants from mice immunized with PBS, inactivated MAP, M506070, or M506070-MYY. Data are presented as mean ± SD (*n* = 5 per group at each time point). Statistical significance was assessed using ordinary two-way ANOVA followed by Dunnett’s multiple-comparisons test, with each vaccine group compared with the PBS control at the corresponding time point. *p* < 0.05 (*), *p* < 0.01 (**), *p* < 0.001 (***), and *p* < 0.0001 (****); ns, not significant.

To characterize vaccine-induced cellular immune responses, splenocytes isolated from separate subsets of immunized mice at weeks 2, 4, and 8 were restimulated ex vivo with the recombinant MAP multi-epitope vaccine protein for 48 h. IFN-γ, IL-17, TNF-α, and IL-2 concentrations in the resulting culture supernatants were subsequently determined by ELISA (Figure 9).

Antigen-restimulated splenocytes from the immunized groups generally produced higher cytokine concentrations than those from the PBS control group. The M506070 group showed antigen-responsive cytokine production, whereas the M506070-MYY group generally showed higher mean cytokine values among the recombinant-protein formulations without reaching statistical significance; this pattern should be interpreted cautiously because MYY was not tested alone.

For IFN-γ, the M506070-MYY group produced higher IFN-γ levels than PBS at the evaluated post-immunization time points. IFN-γ production was also increased in the M506070 group and in the inactivated MAP group, although the response was generally lower than that observed with the MYY-containing recombinant formulation.

A similar pattern was observed for IL-17. The M506070-MYY group showed increased IL-17 production compared with PBS following antigen restimulation, and IL-17 production remained elevated during the observation period; this result was interpreted as a formulation-associated response. The M506070 group also produced detectable IL-17 responses, supporting the intrinsic immunogenicity of the recombinant protein.

For TNF-α, the M506070-MYY group exhibited increased TNF-α production compared with PBS. Increased TNF-α production was also detected in the M506070 and inactivated MAP groups at later time points.

IL-2 production was elevated relative to PBS in the immunized groups at the evaluated time points, with the M506070-MYY formulation showing antigen-responsive IL-2 production throughout the observation period.

This cytokine profile shows that the recombinant MAP multi-epitope protein generated splenocytes capable of responding to antigen restimulation and that the MYY-containing formulation was associated with higher mean readouts in this setting, although the differences between the two recombinant-protein groups did not reach statistical significance. These data support the immunogenicity of the recombinant construct, while leaving protective activity against MAP infection to be tested directly.

### 3.11. Transcriptomic Profiling Reveals Macrophage Responses to the Recombinant Multi-Epitope Vaccine Protein

To further characterize early cellular responses associated with the recombinant vaccine candidate, RAW264.7 macrophages were stimulated with the purified recombinant multi-epitope vaccine protein and subjected to transcriptomic analysis.

**Figure 10:**
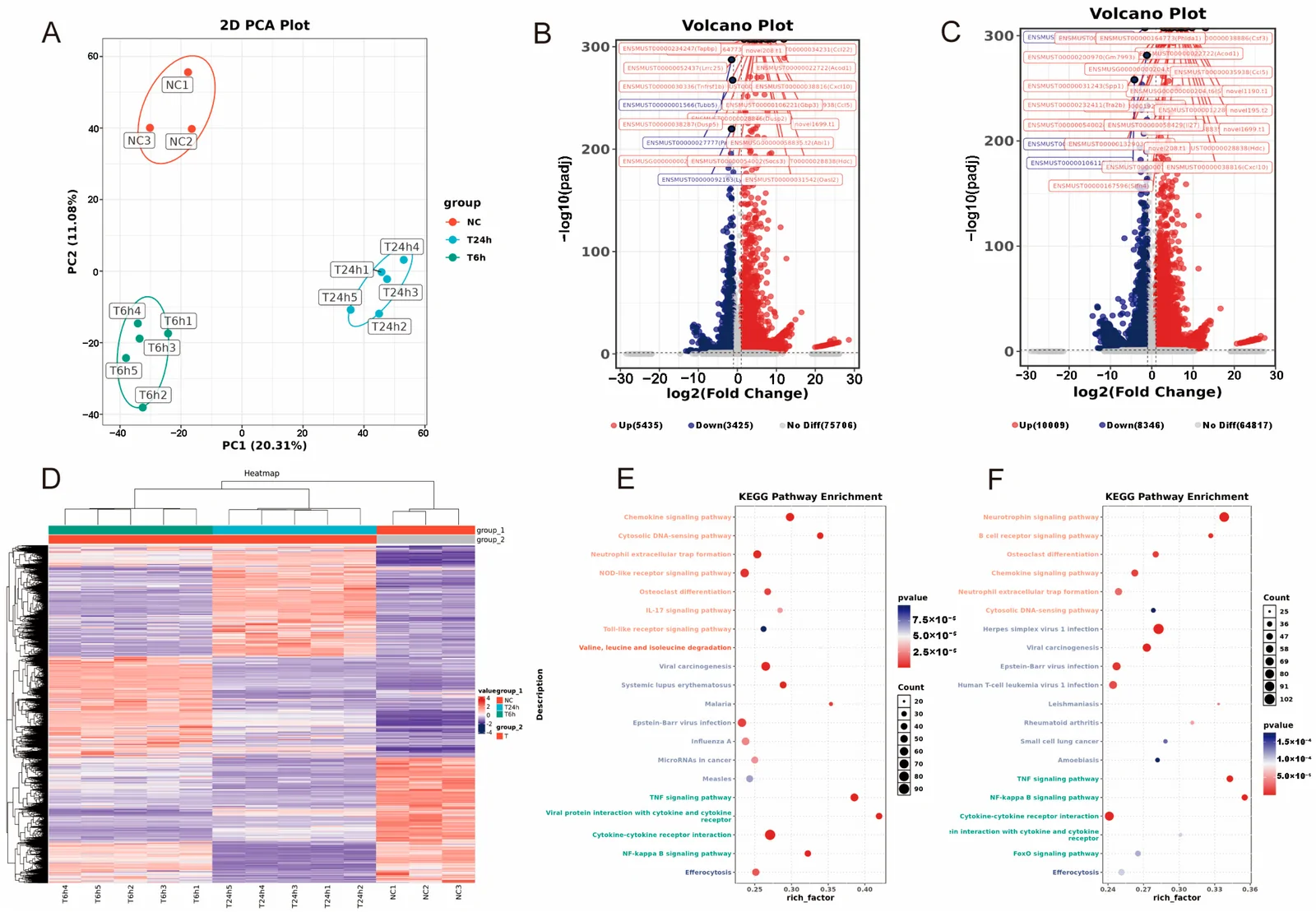
Transcriptomic profiling of RAW264.7 macrophages following recombinant multi-epitope vaccine protein stimulation. (**A**) Principal component analysis (PCA) of transcriptomic profiles from the NC, T6h, and T24h groups. (**B**) Volcano plot showing differentially expressed genes (DEGs) between the NC and T6h groups. (**C**) Volcano plot showing DEGs between the NC and T24h groups. (**D**) Heatmap of differentially expressed genes across all samples. (**E**) KEGG pathway enrichment analysis of DEGs identified at 6 h after recombinant protein stimulation. (**F**) KEGG pathway enrichment analysis of DEGs identified at 24 h after recombinant protein stimulation. Differentially expressed genes were identified using the indicated thresholds, and significantly enriched pathways are ranked according to enrichment significance.

Principal component analysis showed clear separation among the NC, T6h, and T24h groups, while biological replicates within each group clustered closely, indicating distinct transcriptional profiles following recombinant protein stimulation (Figure 10A).

Compared with the NC group, 5435 genes were upregulated and 3425 genes were downregulated at 6 h (Figure 10B). At 24 h, 10,009 genes were upregulated and 8346 genes were downregulated relative to the NC group (Figure 10C). Hierarchical clustering similarly distinguished the NC, T6h, and T24h groups based on their global gene-expression profiles (Figure 10D).

KEGG pathway enrichment analysis showed that differentially expressed genes at 6 h were enriched in several immune- and inflammation-associated pathways, including cytokine-cytokine receptor interaction, NF-κB signaling, TNF signaling, IL-17 signaling, chemokine signaling, Toll-like receptor signaling, and NOD-like receptor signaling (Figure 10E).

At 24 h, cytokine-cytokine receptor interaction, NF-κB signaling, and chemokine signaling remained among the enriched pathways. Additional enriched pathway annotations included B-cell receptor signaling, neurotrophin signaling, and FoxO signaling (Figure 10F).

Recombinant-protein stimulation markedly reshaped the RAW264.7 macrophage transcriptome and enriched immune- and inflammation-related pathway annotations. This molecular profile provides an additional view of the early cellular response to the recombinant protein.

## 4. Discussion

### 4.1. Rational Antigen Selection Provides a Basis for MAP Multi-Epitope Vaccine Development

MAP vaccine development is complicated by the organism’s intracellular lifestyle and its ability to persist in macrophages. These features make it unlikely that a narrow antibody-only response would be sufficient. A candidate vaccine is more likely to be useful if it can support antigen recognition while also promoting cellular responses linked to macrophage activation and T-cell function [3,4,5].

Single-antigen subunit vaccines are more defined, although that precision may also limit immune coverage against a pathogen with several persistence and immune-modulation mechanisms [6].

In this study, seven MAP proteins were selected after screening for predicted antigenicity, immunogenicity, toxicity, allergenicity, and sequence conservation. Several proteins showed high similarity across the examined MAP strains, which supported their use as antigen sources for a construct intended to retain broader strain relevance.

The computational screen should therefore be viewed as a triage step, not as evidence that any selected protein is protective. Its value here was to reduce the search space and provide a defined antigen set that could be carried forward into construct design, recombinant expression, and immunogenicity testing [7].

Beyond the prediction-based criteria, biological diversity among antigen sources was also considered during candidate selection. The seven retained proteins could be broadly grouped into three complementary categories: host-interaction and cell-surface-associated proteins, proteins related to intracellular stress adaptation and homeostasis, and regulatory or less-characterized conserved proteins. This categorization reflects the different biological contexts encountered during MAP infection, including host–cell interaction and entry, adaptation to the intracellular environment, and persistence within macrophages [3,4]. Thus, functional diversity complemented the immunoinformatic ranking criteria and broadened the antigen-source repertoire for multi-epitope construction.

The first category comprised the Mce protein (Q73TV8) and lipoprotein (Q73YG3), which represent host-interaction and cell-envelope-associated antigens. MAP Mce proteins have been implicated in host–cell attachment and invasion, and recent experimental work showed that selected MAP Mce proteins can promote interaction with bovine epithelial and phagocytic cells [35]. Consistently, genome-wide transposon screening in dairy calves identified multiple mce genes and an mce-family lipoprotein as important for MAP persistence in the natural host [36]. MAP lipoproteins also have documented immunological relevance, as individual lipoproteins have elicited cellular and humoral responses in infected or vaccinated animals [37,38], although these findings do not directly validate Q73YG3. The second category included the proteasome beta subunit (Q73YW8) and thioredoxin-like fold domain-containing protein (Q73VK8), representing functions potentially related to intracellular stress adaptation and cellular homeostasis. In mycobacteria, the proteasome contributes to resistance to reactive nitrogen intermediates, while thioredoxin systems participate in thiol-disulfide exchange and redox homeostasis [39,40]. These findings provide a biological rationale for including proteins associated with host interaction and intracellular stress responses, while their reported functions should not be directly assigned to the corresponding MAP proteins without experimental validation.

The third category comprised the probable regulatory protein B5LU12, the DUF732 domain-containing protein Q73Y71, and the uncharacterized protein Q73Y48. Direct functional evidence for these proteins in MAP remains limited; therefore, their biological roles should not be inferred beyond their current annotations. Their retention instead reflects the broader advantage of reverse-vaccinology approaches, in which conserved proteins satisfying predefined antigenicity and safety criteria can be considered even when they are not established virulence factors [7]. In the present study, these proteins passed the integrated computational screening, while B5LU12 and Q73Y48 also showed high conservation among the examined MAP strains. Taken together, the three categories provided complementary antigen sources spanning host interaction, intracellular adaptation, and less-characterized conserved proteins, thereby reducing reliance on a single biological pathway or protein class [6,7]. Nevertheless, biological relevance, sequence conservation, and predicted antigenicity do not establish protective efficacy, and the contribution of individual proteins and epitopes requires validation in ruminant immune systems and MAP challenge models.

In addition to inducing effective immune responses, compatibility with existing diagnostic systems represents an important consideration for next-generation MAP vaccine development. Whole-cell MAP vaccines contain broad repertoires of mycobacterial antigens and may introduce shared antigenic components that complicate interpretation of bovine tuberculosis diagnostic assays. In contrast, the defined composition of multi-epitope vaccines provides an opportunity to reduce unintended overlap with diagnostic targets. In the present study, M506070 showed no substantial sequence similarity to ESAT-6, CFP-10, MPB70, or MPB83, and the only database-level homologs identified were L7/L12 proteins from other mycobacterial species, consistent with the intended immunostimulatory module (Section 3.3, Table 1). Taken together, these findings support the potential DIVA-compatible characteristics of M506070; however, experimental validation using MAP-infected and vaccinated animals will be required to determine whether the candidate can reliably differentiate vaccination-induced responses from natural infection.

### 4.2. Multi-Epitope Vaccine Design Enables Broad Immunological Targeting

We used the multi-epitope format to place different predicted immune targets into a single recombinant protein. The final construct contained seven MHC class II-restricted HTL epitopes, five B-cell epitopes, and ten MHC class I-restricted CTL epitopes, combining regions intended to support antibody recognition with regions predicted to engage cellular immune pathways.

This design is relevant to MAP because the pathogen is intracellular. Antibody responses may contribute to antigen recognition, opsonization, and antigen uptake, but T-cell-associated responses are also expected to be important for anti-mycobacterial immunity. Including CTL and HTL epitopes therefore allowed the construct to address more than one arm of the immune response [3,4,9].

The linker design was another important part of the construct. GPGPG, AAY, EAAAK, and KK linkers were used to separate epitope groups and functional regions, reducing the likelihood that adjacent peptides would interfere with antigen processing or the predicted structure of the recombinant protein [7].

Prediction alone cannot show that these epitopes are processed, presented, or displayed in the expected conformation. The subsequent detection of antigen-specific antibodies and cytokine-producing splenocytes indicates that the expressed protein was immunogenic as a whole, although the contribution of individual epitopes remains unresolved [7,10].

### 4.3. Structural Modeling and Molecular Docking Characterize the Designed Construct

We included structural analysis because peptide order and linker placement can affect the folding and accessibility of engineered multi-epitope proteins. After refinement, the proportion of residues in the most favored regions of the Ramachandran plot increased from 79.6% to 94.8%, and the ProSA Z-score changed from −5.12 to −5.56. These changes justified using the refined model for the docking step.

The refined structure was docked with bovine TLR1, TLR2, and TLR6 models. The TLR6-vaccine complex had the most favorable HDOCK score, followed by TLR2 and TLR1. Interface analysis also identified predicted hydrogen bonds, salt bridges, and large contact surfaces in the three receptor-vaccine complexes.

TLR2-containing receptor complexes are relevant to innate recognition of microbial and mycobacterial components, so these docking analyses provided a structural view of possible receptor compatibility. These results should not be read as binding-affinity measurements or as direct evidence of receptor activation. Instead, they point to receptor interactions that can be tested in later functional experiments [9].

The macrophage transcriptomic data also showed enrichment of Toll-like receptor-related pathways after recombinant-protein stimulation. This observation is consistent with innate immune activation, but it does not identify the specific receptor responsible for the response. TLR-blocking assays or receptor-deficient cell models would be needed to test whether TLR1, TLR2, or TLR6 participates directly [9].

### 4.4. The Multi-Epitope Vaccine Candidate Induces Coordinated Humoral and Cellular Immune Responses

For an intracellular pathogen such as MAP, antibody production alone is not the most informative early readout; cellular responses also need to be considered. The recombinant multi-epitope formulation induced antigen-specific IgG responses and increased cytokine production after splenocyte restimulation, indicating that the expressed construct was recognized immunologically after vaccination.

Beyond measuring antibody magnitude, the antigen-recognition profile provided additional information regarding the specificity of vaccine-induced humoral responses. In this study, sera from M506070-immunized and M506070-MYY-immunized mice showed strong recognition of recombinant M506070 protein and detectable binding to MAP lysate, indicating that antibodies induced by the multi-epitope construct could recognize both the designed antigen and components present in MAP lysate. In contrast, limited reactivity was observed when PPD was used as the coating antigen, suggesting that the antibody response generated by M506070 was more closely associated with the defined vaccine antigen rather than broad recognition of conventional mycobacterial diagnostic preparations. These findings support the concept that a defined multi-epitope vaccine may induce MAP-associated immune recognition while maintaining a more restricted antigenic profile compared with whole-cell vaccine preparations [6].

Total antigen-specific IgG increased after immunization, and both IgG1 and IgG2a subclasses were detected. The IgG2a/IgG1 ratio remained close to 1 across the evaluated time points, suggesting that the response was not dominated by either subclass. Because mouse IgG subclasses provide only an indirect indication of helper T-cell bias, these data are best interpreted alongside the cytokine results rather than as a standalone measure of Th polarization.

The cytokine data provided clearer evidence of antigen-responsive cellular activity. Splenocytes collected four weeks after immunization showed increased production of IFN-γ, IL-2, TNF-α, and IL-17 following ex vivo stimulation with recombinant M506070 protein or MAP lysate, whereas PPD stimulation induced only limited responses. The strongest cytokine responses were observed after stimulation with recombinant M506070, indicating that vaccination generated antigen-specific cellular recall responses against the designed construct. Responses induced by MAP lysate stimulation further suggested recognition of components present in MAP lysate, whereas the relatively limited response pattern following PPD stimulation was consistent with the defined antigen composition of the multi-epitope vaccine. Together, these results indicate that M506070 vaccination activated both antigen-specific cellular immunity and MAP-associated immune recognition. This pattern is compatible with recall responses involving Th1- and Th17-associated features, both of which are relevant to macrophage activation and inflammatory regulation during mycobacterial infection [5,8,41].

The M506070 group was included to establish the intrinsic immunogenicity of the recombinant construct, whereas MYY was evaluated as an exploratory external formulation component to determine whether additional immune enhancement could be achieved beyond the intrinsic immunostimulatory design of the construct. Additional adjuvant formulations are commonly explored for recombinant subunit vaccines because purified protein antigens may provide limited innate immune stimulation [42,43]. Higher mean readouts in the M506070-MYY group suggested a possible formulation-associated trend, although statistically significant differences between the two recombinant-protein groups were not observed; this observation should therefore be regarded as exploratory rather than definitive evidence of MYY-specific adjuvant activity.

These immune responses justify further testing of the construct. Whether they reduce MAP replication, tissue colonization, lesion development, or shedding will require controlled MAP challenge experiments [6].

### 4.5. Transcriptomic Profiling Provides Insights into Macrophage Responses to the Recombinant Vaccine Candidate

The RAW264.7 RNA-seq experiment examined how macrophages responded shortly after exposure to the recombinant protein. PCA separated the NC, T6h, and T24h groups, and differential expression analysis showed extensive transcriptional changes at both time points. This pattern indicates a strong response to the recombinant protein in RAW264.7 macrophages under the tested conditions.

KEGG enrichment analysis identified immune- and inflammation-associated pathways, including cytokine-cytokine receptor interaction, NF-κB signaling, TNF signaling, IL-17 signaling, chemokine signaling, Toll-like receptor signaling, and NOD-like receptor signaling. These pathways are consistent with macrophage activation, inflammatory signaling, and cytokine-mediated cell communication [9,33,34].

Some enrichment results require careful interpretation. For example, B-cell receptor signaling was annotated at 24 h, although the experiment used RAW264.7 macrophages rather than B cells. This annotation probably reflects overlap among genes shared by multiple KEGG pathways rather than direct evidence of B-cell activation [33,34].

The transcriptomic findings also align partly with the mouse immunogenicity results. Enrichment of cytokine-, TNF-, and IL-17-associated pathways in stimulated macrophages parallels the increased TNF-α and IL-17 production observed from antigen-restimulated splenocytes. The two systems are not mechanistically equivalent, but the overlap supports the view that the construct can engage immune-related inflammatory networks [9,41].

Because selected RNA-seq findings were not independently validated at the transcript or protein level, the transcriptomic data should be considered an exploratory response signature. Follow-up work should test specific genes and pathways by qPCR, cytokine assays, receptor blocking, or loss-of-function approaches.

### 4.6. Potential Applications and Limitations

This study links antigen selection, construct design, structural analysis, recombinant protein expression, mouse immunogenicity testing, and macrophage transcriptomics in an early-stage evaluation of a MAP vaccine candidate. The main practical advantage of the multi-epitope approach is that it uses defined antigenic regions rather than the broad and less interpretable antigen mixture present in whole-cell preparations.

Several limitations should be considered.

First, the MHC-restricted epitopes were predicted using bovine BoLA alleles, whereas immunogenicity was tested in BALB/c mice. The mouse experiment therefore supports overall recombinant-protein immunogenicity but does not validate BoLA-restricted presentation of individual CTL or HTL epitopes. Direct testing in bovine or ovine immune cells would be a more appropriate next step for evaluating whether the predicted epitopes are processed and presented as intended [8,15].

Second, the study measured immunogenicity rather than protection. The antibody and cytokine responses observed here do not directly address whether vaccination can reduce MAP burden, tissue pathology, disease progression, or bacterial shedding. Those questions require MAP challenge experiments in an appropriate infection model [6].

Third, MAP primarily affects ruminants, whereas the preliminary immunization work used mice. Differences in MHC repertoire, immune physiology, infection kinetics, and vaccine responsiveness may influence both epitope recognition and the overall response to the recombinant construct. Testing in ruminant immune cells and, ultimately, target animals will be important for judging biological relevance [1,8].

Fourth, although an MYY-free recombinant-protein group was included, an adjuvant-only group was not available. Moreover, MYY was purchased from Beijing Centrebio Biology Co., Ltd. (Beijing, China). The formulation is the subject of a patent application by the manufacturer, and its exact active components were not fully defined in the present work. Therefore, the study could describe formulation-associated differences in recombinant-protein responses but could not independently determine whether MYY alone induced nonspecific immune activation or contributed directly to the observed differences.

Fifth, the transcriptomic experiment used a macrophage cell line stimulated in vitro. RAW264.7 cells are useful for detecting early macrophage-associated responses, but they cannot reproduce the complexity of vaccination or MAP infection in vivo. Residual nonspecific inflammatory stimulation also cannot be excluded completely, even though endotoxin removal and LAL testing were performed.

Finally, the study did not determine which individual epitopes or antigen-source proteins contributed most strongly to the observed immune responses. Peptide-specific T-cell assays, antibody mapping, receptor-blocking experiments, and targeted pathway validation would help refine the construct and identify the most useful components.

Overall, the construct advanced beyond prediction: it was expressed as a recombinant protein, induced antigen-specific antibody responses, and generated splenocyte cytokine responses after antigen restimulation. The next step is evaluation in MAP challenge models and in immune cells or animals from ruminant hosts.

## 5. Conclusions

This study designed and experimentally evaluated a MAP multi-epitope recombinant vaccine candidate. The construct was successfully expressed, induced antigen-specific antibody responses, and generated splenocyte cytokine responses after antigen restimulation in mice. The MYY-containing formulation produced numerically higher immune-response readouts in several assays without reaching statistical significance, but this observation should be interpreted cautiously because an MYY-only group was not included and the adjuvant formulation was not fully characterized in this study. Further work should validate epitope presentation in ruminant immune cells and test protective efficacy in MAP challenge models and target animals.

## Figures and Tables

**Table 1:** Sequence similarity between the M506070 multi-epitope vaccine construct and bovine tuberculosis diagnostic antigens.

Diagnostic Antigen	Sequence Identity (%)	Longest Continuous Identical Sequence in Full Length (aa)	Longest Continuous Identical Sequence Within Epitope Regions (aa)
ESAT-6	<30	4	≤4
CFP-10	<30	3	≤4
MPB70	<30	6	≤4
MPB83	<30	5	≤4

## Data Availability

The RNA-seq data generated in this study have been deposited in the Gene Expression Omnibus (GEO) database under accession number GSE345542. Other data supporting the findings of this study are available from the corresponding authors upon reasonable request.

## Supplementary Materials

The following supporting information can be downloaded at: https://www.mdpi.com/article/10.3390/microorganisms14102188/s1, Table S1: Summary of immunoinformatics tools, parameters, and selection thresholds used in this study; Table S2: Selected Mycobacterium avium subsp. paratuberculosis proteins used for multi-epitope vaccine design; Table S3: Protein homology and comparative sequence analysis across different MAP strains; Table S4: Selected HTL, CTL, and B-cell epitopes used for construction of the multi-epitope vaccine candidate.
