# Supplementary material for: Immunoinformatics-Guided Design, Immunological Evaluation, and Macrophage Transcriptomic Profiling of a Multi-Epitope Vaccine Candidate Against Mycobacterium avium subsp. paratuberculosis
Source: Microorganisms. 2026 Sep 30;14(10):2188. doi: 10.3390/microorganisms14102188 (PMC13707567; doi:10.3390/microorganisms14102188)
Supplement: Supplementary file 1 [file microorganisms-14-02188-s001.zip › microorganisms-4559322-supplementary.pdf]

Supplementary Materials

Table S1. Summary of immunoinformatics tools, parameters, and selection thresholds used in this study.

Analysis step	Tool/server	Version/model	Main input	Selection threshold or ranking criterion	Output used for final decision
Protein antigenicity	VaxiJen	v2.0; bacterial model	MAP protein sequences	Score-based prioritization	Antigenicity score
Toxicity prediction	ToxinPred	Default parameters	MAP protein sequences or peptides	Predicted non-toxic	Toxicity status
Allergenicity prediction	AllerTOP	v2.0; default parameters	MAP protein sequences or peptides	Predicted non-allergenic	Allergenicity status
B-cell epitope prediction	ABCpred	16-aa peptide setting	Candidate antigen sequences	Weighted score: ABC score × 40% + immunogenicity × 30% + solubility probability × 30%	ABC score and integrated ranking score
MHC-I epitope prediction	NetMHC	v4.0	Candidate antigen sequences and BoLA-I alleles	Weighted score: class I immunogenicity × 40% + immunogenicity × 30% + solubility probability × 30%; followed by construct-level assessment	Binding prediction/rank and integrated ranking score
MHC-II epitope prediction	NetMHCIIpan	v4.3	Candidate antigen sequences and BoLA-DRB3 alleles	Default strong-binder criterion: %Rank_EL < 1.0%; followed by integrated weighted ranking	Binding prediction/rank
IFN-γ-inducing potential	IFNepitope	Default settings	HTL candidate peptides	Weighted score component for HTL ranking	IFN-γ score
Solubility prediction	ProtSol	Default output	Candidate peptides	Ranked by solubility probability and included in weighted score	Solubility probability
Sequence conservation	BLASTp	Default alignment output	Candidate proteins and homologs	Score-based prioritization using sequence similarity among examined MAP strains	Sequence similarity

Table S2. Selected Mycobacterium avium subsp. paratuberculosis proteins used for multi-epitope vaccine design.

Protein	UniProt ID	Size (AA)	Homology	Toxicity	Allergenicity	Immunogenicity Score
Proteasome subunit beta	Q73YW8	303	None	None	None	1.027
Probable regulatory protein	B5LU12	207	None	None	None	1.0294
Uncharacterized protein	Q73Y48	136	None	None	None	1.0292
DUF732 domain-containing protein	Q73Y71	166	None	None	None	1.0194
Thioredoxin-like fold domain-containing protein	Q73VK8	255	None	None	None	1.0328
Mce protein	Q73TV8	213	None	None	None	1.0356
Lipoprotein	Q73YG3	247	None	None	None	1.0287

Table S3. Protein homology and comparative sequence analysis across different MAP strains.

Protein	Strain	Comparative Protein	Sequence similarity (%)
Proteasome subunit beta	GCA_003815795.1	AZA71377.1	100
Probable regulatory protein		AZA69061.1	99.5
Uncharacterized protein		AZA71129.1	100
DUF732 domain-containing protein		AZA71150.1	84.3
Thioredoxin-like fold domain-containing protein		AZA70317.1	100
Mce protein		AZA69752.1	91.8
Lipoprotein		AZA72181.1	74.1
Proteasome subunit beta	GCA_003957335.1	AZP81443.1	100
Probable regulatory protein		AZP79560.1	98.1
Uncharacterized protein		AZP81673.1	99.3
DUF732 domain-containing protein		AZP81212.1	84.3
Thioredoxin-like fold domain-containing protein		AZP80287.1	100
Mce protein		AZP83395.1	91.8
Lipoprotein		AZP83729.1	74.1
Proteasome subunit beta	GCA_043590545.1	XII80174.1	100
Probable regulatory protein		XII77872.1	99.5
Uncharacterized protein		XII79931.1	100
DUF732 domain-containing protein		XII79950.1	84.3
Thioredoxin-like fold domain-containing protein		XII79109.1	100
Mce protein		XII78551.1	91.4
Lipoprotein		XII80037.1	78.9

Table S4. Selected HTL, CTL, and B-cell epitopes used for construction of the multi-epitope vaccine candidate.

Epitope Sequence	ABC Score	Class I Immunogenicity	IFN-γ-inducing score	Immunogenicity score	Solubility probability
B-cell Epitopes					
AQSGPASSSGGTGQLP	0.83	NA	NA	1.7509	97%
GGSQMTTGTRSQLRVP	0.96	NA	NA	1.5501	90%
AAKIRATPTIKINGED	0.84	NA	NA	1.4973	91%
GDRRSTQGNMIAGRDV	0.83	NA	NA	1.6581	88%
APSETARTAADIRASD	0.89	NA	NA	1.3788	95%
MHC-I Epitopes					
SRGEPSIAW	NA	0.0804	NA	0.8866	97%
STPDALVGK	NA	0.10102	NA	1.0508	93%
EIVGDIPGL	NA	0.20482	NA	0.7186	88%
GSIDILVAL	NA	0.24238	NA	0.6612	86%
AAREDAVAL	NA	0.23368	NA	0.6397	85%
AEWEVESAI	NA	0.19034	NA	0.6796	80%
TGPTEGPAI	NA	0.17836	NA	1.0906	72%
LALWGWPLK	NA	0.44496	NA	1.94	70%
RGPERVVTA	NA	0.24393	NA	0.6206	67%
AAIAAVLVL	NA	0.17429	NA	0.5775	63%
MHC-II Epitopes					
PAYRQSVSASVSASS	NA	NA	0.95625314	1.0019	98%
DADSAVRVAIEALYD	NA	NA	0.14583643	0.6309	94%
SGKYIAKVTGEAAAA	NA	NA	0.28293539	0.5159	92%
SVDKDLAAARDRLTG	NA	NA	0.23784869	0.5566	91%
GAGYLRWSAGSADDL	NA	NA	0.26077409	0.7876	98%
AVALLSYRADSVDKD	NA	NA	0.02433092	0.5739	91%
PGGVVLAGDRRSTQG	NA	NA	0.091193189	0.7248	76%
